# Advanced Guareschi–Thorpe synthesis of pyridines in green buffer, and pH-controlled aqueous medium with ammonium carbonate†

**DOI:** 10.1039/d3ra04590k

**Published:** 2023-08-21

**Authors:** Fatemeh Tamaddon, Sajedeh Maddah-Roodan

**Affiliations:** a Department of Chemistry, Faculty of Science, Yazd University Yazd 89195-741 Iran ftamaddon@yazd.ac.ir sajedeh.maddah@stu.yazd.ac.ir

## Abstract

Hydroxy-cyanopyridines are easily synthesized *via* an advanced version of the Guareschi–Thorpe reaction by either three-component condensation of alkyl cyanoacetate or cyanoacetamide with 1,3-dicarbonyls and ammonium carbonate in an aqueous medium. Reactions proceed productively to give the desired products in high yields. Simple mechanistic monitoring showed the role of (NH_4_)_2_CO_3_ as both a nitrogen source for the pyridine ring and the reaction promoter. This new multicomponent approach for pyridine synthesis is inexpensive, user-friendly, and eco-friendly, while green buffer conditions, versatility, precipitation of products in the reaction medium, and simple work-up are extra advantages.

## Abstract 2

Hydroxy-cyanopyridines are easily synthesized *via* an advanced version of the Guareschi–Thorpe reaction by three-component condensation of alkyl cyanoacetate or cyanoacetamide with 1,3-dicarbonyls and ammonium carbonate in aqueous medium.

## Graphical abstract

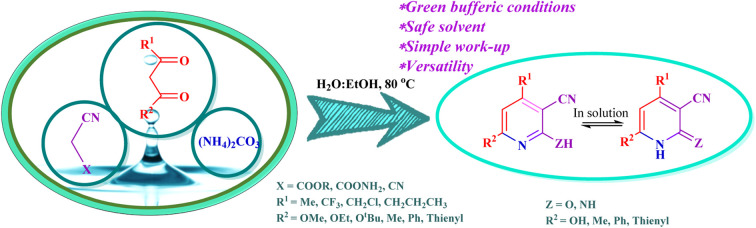

## 1 Introduction

Pyridine derivatives, which are extensively used as the solvent, catalyst, and base,^1–3^ constitute the skeletal part of the critical bio-organic medicals and are synthon precursors for vitamins, pharmaceuticals, food flavors, paints, dyes, adhesives, herbicides, insecticides, and rubbers.^4,5^ Despite the development of various methods for the synthesis of pyridines,^6,7^ the Guareschi–Thorpe (GT) method that gives hydroxy pyridines or their pyridone tautomer has rarely been investigated.^8–11^ The importance of this reaction is due to the bioactivity of the pyridone/hydroxypyridine tautomer pairs,^12–14^ in which their pyrimidine aza-analogs have an important roles in the structure of DNA nucleotides and related biomolecules. Besides, these tautomer pairs are critical intermediates for the synthesis of pharmaceuticals^15^ and clinically used medicines,^16^ including pirfenidone,^17^ ciclopirox,^18^ and huperazine.^19^ The original GT reaction^8^ is a usual method for the Hantzsch synthesis of pyridines^20^ and a [3 + 2 + 1] synthetic strategy to provide the hydroxy pyridines *via* the three-component reaction of a β-diester with ammonium acetate and ethylcyanoacetate.^21^ Classical GT reaction is a multi-component reaction (MCR) run by refluxing the excess of cyanoethylacetate, diethylmalonate, and ammonium acetate in an azeotropic mixture of acetic acid, benzene, and water for 45 hours.^22^ A two-component [3 + 3] variant of the GT reaction for condensation of β-dicarbonyl compounds and cyanoacetamide, in not so high yields, has also been reported in organic solvents.^23^ To avoid the negative impacts of the volatile organic solvents (VOSs) and corrosive catalysts, the development of more green water-based versions of GT reaction is highly anticipated.

**Scheme 1:**
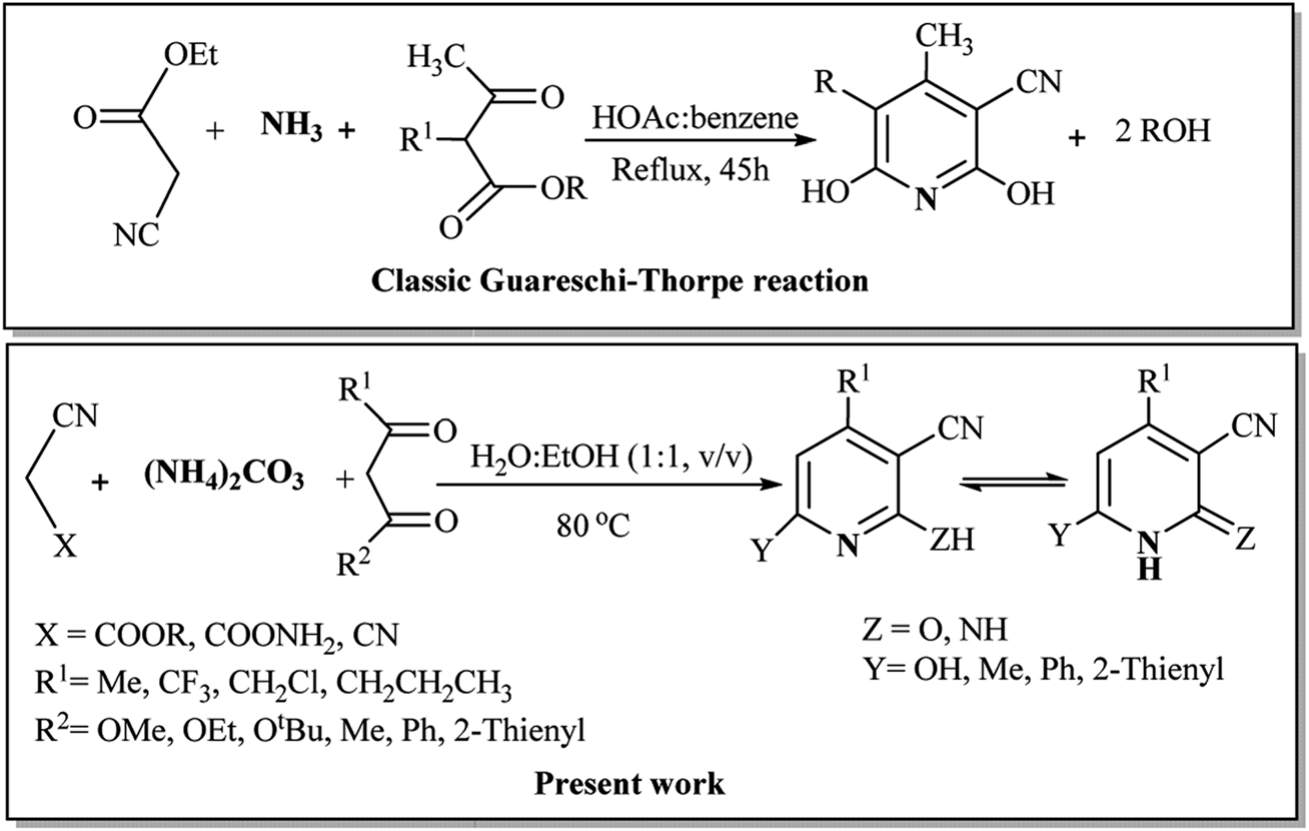
Guareschi–Thorpe reaction in water using ammonium carbonate.

Generally, the synthetic reactions need solvent, while most of organic solvents are volatile, toxic, flammable, and their reusing needs energy consumption. Green chemistry aims to minimize the VOS hazards in chemical processes by replacing them with more sustainable alternatives.^24^ Water is the greenest alternative for the VOSs.^25^ Thus, carrying out the organic reactions in water has been extensively developed.^26^ Water-based organic reactions are highly desirable due to the advantages of the zero E-factor of water, polarity, hydrogen-bonding network, and hydrophobic interactions with organic materials to decrease the activation volume-change (Δ*V*)^#^ and enhance the reaction rate.^27^ Additionally, a series of water-based organic reactions accelerated by microwave^28–30^ or ultrasound irradiation^31^ occurred within minute scale times instead of days. Combination of the water-based organic synthesis^32^ with the multi-component reactions^33,34^ provided water-based MCRs^35^ as a highlight division of green chemistry to produce the complex bioorganic molecules. Typically, water-based MCRs have developed as recent post-methods for the synthesis of various heterocyclic compounds.^36,37^ We have also developed Michael, Mannich, Biginelli, Hantzch, and other MCRs for the synthesis of heterocycles in water.^38–43^ Catalyst-free MCRs in aqueous media are advanced promising examples of an ideal reaction for the synthesis of organic compounds, especially when the final product precipitates in water. Due to the importance of the hydroxy pyridine/pyridone derivatives and water-based organic reactions, we reported herein a new green version of GT reaction in water. In this method, ammonium carbonate is either a non-toxic nitrogen source or a pH-controlled agent for water-based synthesis of hydroxy pyridines under thermally or ultrasound conditions (Scheme 1).

## 2 Results and discussion

In our preliminary experiment, the three-component GT reaction of ethyl acetoacetate, ethyl cyanoacetate, and ammonium acetate performed in refluxed azeotropic mixture of H_2_O : HOAc for 24 h. Upon cooling to room temperature, no precipitated product, and reaction analysis indicated the formation of a mixture of products together with the remaining starting materials. Based on our previous experiences on the selection of nitrogen source for heterocycles^39–44^ and to reach a better yield, we compared the GT synthesis of 2,6-dihydroxy-3-cyano-4-methyl pyridine (1a) by three-component reaction of ethyl acetoacetate, ethyl cyanoacetate, and various ammonium salts in an aqueous media (Table 1).

**Table 1:** Optimization of nitrogen source in GT model reaction^a^

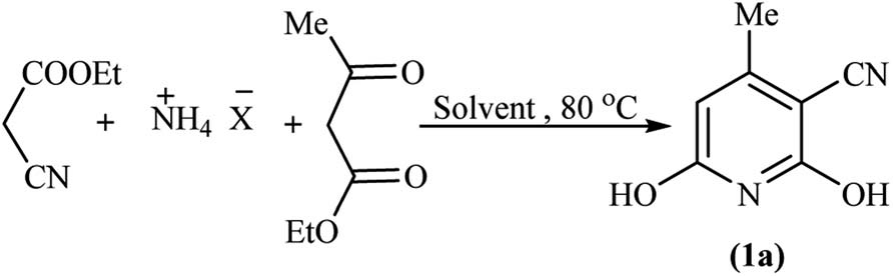				
Entry	Nitrogen source (mmol)	Solvent (mL)	Time (h)	Yield (%)
1	NH_4_Cl (1)	H_2_O	8	32
2	NH_3_ (1)	H_2_O	45	40
3	NH_4_OAc (1)	H_2_O	24	68
4	NH_4_NO_3_ (1)	H_2_O	10	38
5	NH_4_Cl/Na_2_CO_3_ (1)	H_2_O	6	70
7	(NH_4_)_2_CO_3_ (1)	H_2_O	5	75
8	(NH_4_)_2_CO_3_ (0.5)	H_2_O	10	72
9	(NH_4_)_2_CO_3_ (1.5)	H_2_O	5	90
10	(NH_4_)_2_CO_3_ (2)	H_2_O	5	93
11	(NH_4_)_2_CO_3_ (2)	H_2_O : EtOH (1 : 1, v/v)	4	96
12	(NH_4_)_2_CO_3_ (2)	EtOH	5	85
13	(NH_4_)_2_CO_3_ (2)	H_2_O : HOAc (1 : 1)	24	53
14	(NH_4_)_2_CO_3_ (2)	HOAc	24	50
15^b^	(NH_4_)_2_CO_3_ (2)	H_2_O : EtOH (1 : 1, v/v)	4	95

a Reaction conditions: ethyl cyanoacetate (1 mmol), ethyl acetoacetate (1 mmol), solvent (2 mL), nitrogen source, 80 °C.

b Reaction in 20 mmol scale.

As results show, the maximum yield of hydroxy pyridine 1a is due to the reactions run with (NH_4_)_2_CO_3_ (entries 7–12), although only 68% yield of 1a was isolated after 24 h by the same set-upped reaction with ammonium acetate at 80 °C (entry 3). However, the best yield of product 1a was obtained from the reaction run with 2 mmol ammonium carbonate and 1 : 1 volume ratio of H_2_O : EtOH as solvent (entry 11), which in one mmol of (NH_4_)_2_CO_3_ is nitrogen source and the other mol acts as the reaction promoter. The lower yield of reaction in EtOH is due to the sublimation of (NH_4_)_2_CO_3_ (entry 12). A 20 mmol scale-up of this one-pot three-component GT reaction was also successful to give the high yield of product 1a under the optimized conditions (entry 15).

**Scheme 2:**
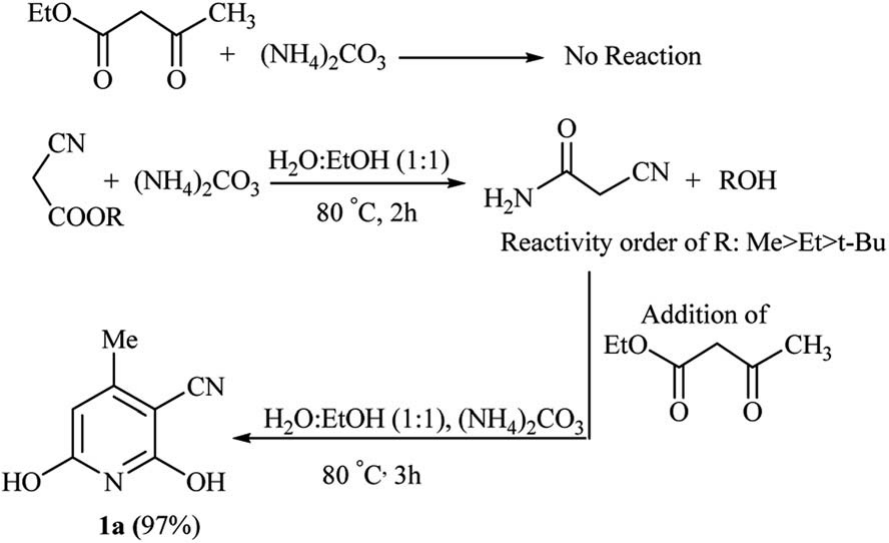
Experimental investigation on the possible mechanism.

Mechanistically, we assumed the reaction of (NH_4_)_2_CO_3_ with ethyl cyanoacetate in an aqueous medium of reaction for the *in situ* production of cyanoacetamide as an intermediate. To clarify the details, we designed two individual reactions between (NH_4_)_2_CO_3_ and the other starting materials in water, so ethyl acetoacetate was left unchanged and alkyl cyanoacetate reacted with the released ammonia from (NH_4_)_2_CO_3_ to give the cyanoacetamide. Comparative condensation of various alkyl cyanoacetates with ammonium carbonate showed the reactivity order of methyl cyanoacetate > ethyl cyanoacetate >> *tert*-butyl cyanoacetate, in agreement with the steric factor of alkyl groups (Scheme 2).

**Scheme 3:**
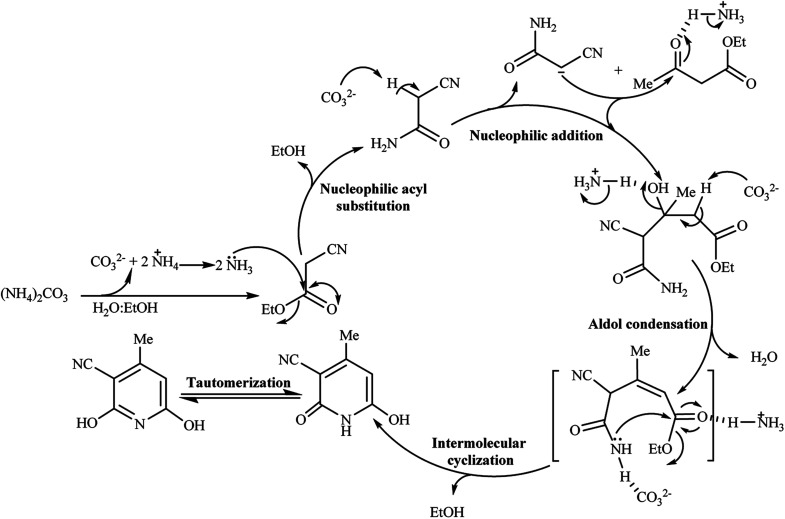
Proposed mechanism for the roles of (NH_4_)_2_CO_3_ in GT reaction.

Following the above reaction of alkyl cyanoacetates with ammonium carbonate by subsequent addition of ethyl acetoacetate led to product 1a again, although with a 5% higher yield for methyl cyanoacetate at a shorter reaction time. From a mechanistic standpoint, ammonium carbonate releases ammonia and also gives carbonic acid, which both can possibly promote the reaction. Bubbling of CO_2_ at beginning the reaction confirmed decomposition of possibly formed carbonic acid at reaction temperature (80 °C). Thus synthetic process initiates by aminolysis of cyanoacetic ester to cyanoacetamide, which undergoes an aldol condensation with the β-ketoester to give a 1,5-dicarbonyl intermediate that finally cyclizes to afford the corresponding pyridine in high yield and purity (Scheme 3).

The generality of the (NH_4_)_2_CO_3_-catalyzed GT reaction was demonstrated by the synthesis of cyano hydroxypyridines (1a–1i) *via* the GT reaction of ethyl cyanoacetate, (NH_4_)_2_CO_3_, and various β-ketoesters or 1,3-diketones under the optimized conditions in 1 : 1 volume ratio of H_2_O : EtOH (Table 2).

**Table 2:** GT reaction of ethyl cyanoacetate, (NH_4_)_2_CO_3_, and 1,3-dicarbonyls^a^

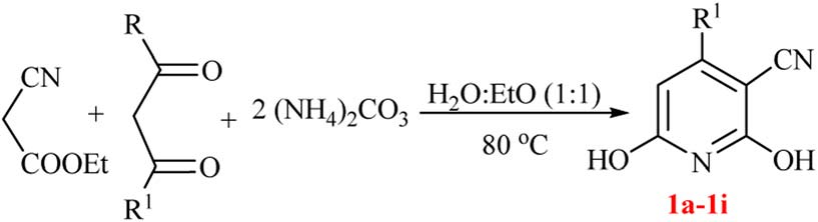					
Entry	R	R^1^	Product	Time (h)	Isolated yield (%)
1	CH_3_	OEt	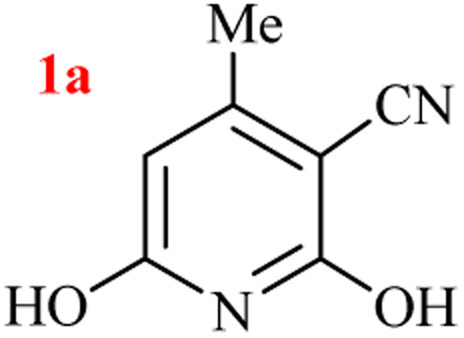	4	96
2	CH_3_	OMe	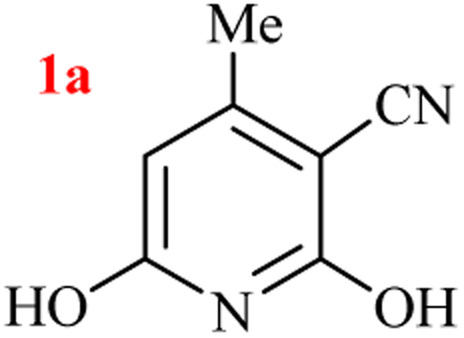	3.5	98
3	CH_3_	O-*t*Bu	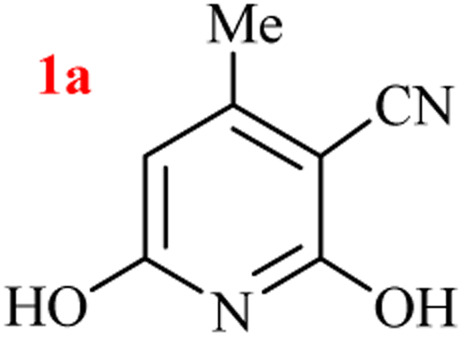	8	85
4	Ph	OEt	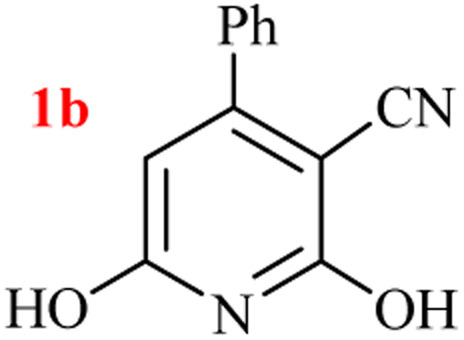	7	93
5	CF_3_	OEt	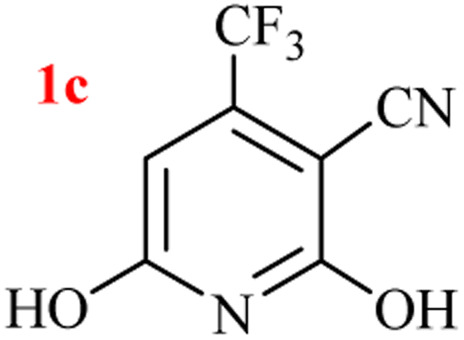	2.5	95
6	CH_2_Cl	OEt	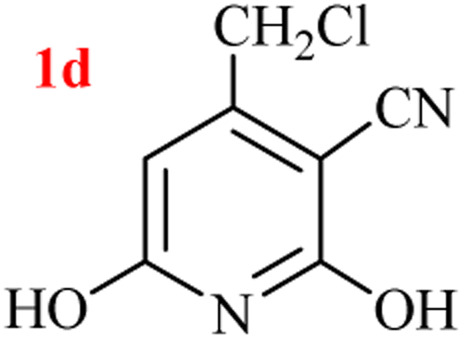	9	90
7	*n*-Propyl	OEt	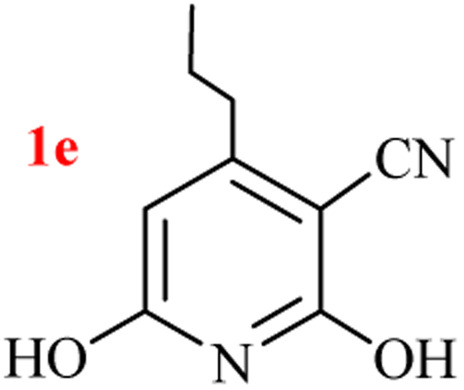	7	85
8	Me	Me	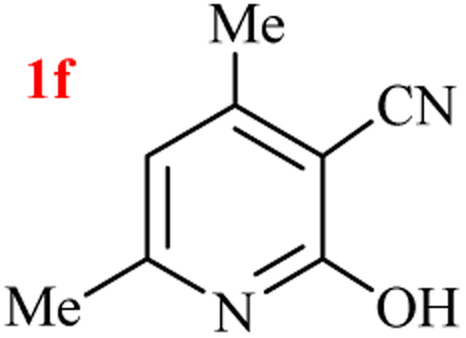	5	90
9	Me	Ph	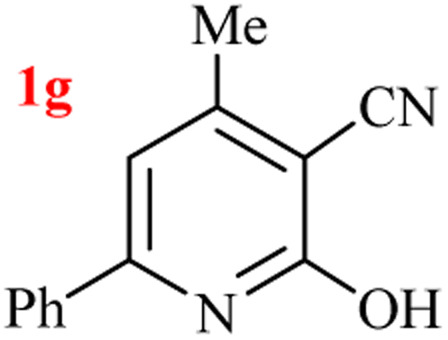	4	87
10	CF_3_	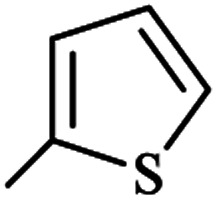	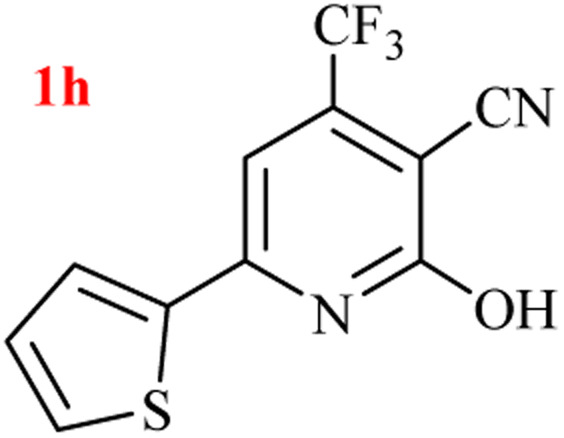	4	84
11	CF_3_	Me	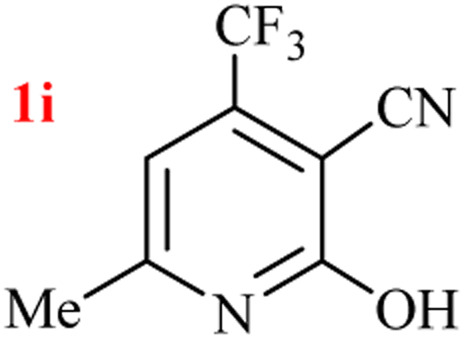	3	90

a Reaction conditions: ethyl cyanoacetate (1 mmol), 1,3-dicarbonyl (1 mmol), (NH_4_)_2_CO_3_ (2 mmol), H_2_O : EtOH (1 : 1), 80 °C.

According to the results, all reactions moved along outstandingly by green conversion of starting materials to high yielding products with no noticeable side product observed by using (NH_4_)_2_CO_3_ as dual ring nitrogen source and reaction promoter. For 2,6-dihydroxy-4-propylpyridine-3-carbonitrile (1e), the FT-IR and ^1^H NMR were satisfactory, but some duplicated peaks in ^13^C NMR spectrum (125 MHz, DMSO-d_6_) may be due to the increase in pyridone tautomer in pyridone/hydroxypyridine tautomer pairs of this 4-aliphatic substituted product (see the ESI†). However, this protocol was suitable for the substituted β-ketoesters (entries 1–7), and 1,3-diketones (8–11). Reducing of reaction times for 1,3-diketones *versus* β-ketoesters can be attributed to the higher electrophilic property and reactivity of the ketone carbonyl group than carbonyl of ester, whereas the electron-withdrawing group of CF_3_ dramatically accelerated the rate of both reaction types (entries 5, 10, and 11) (see also the ESI† for details).

Aqueous solutions of (NH_4_)_2_CO_3_ are well-known powerful buffer solutions with pH = 6.5–7.5. Thus, ammonium carbonate serves two functions in this GT reaction. It behaves either as a mild basic buffer to catalyze the condensation of β-ketoester with ethylcyanoacetate or as a nitrogen source for the resultant pyridine ring, especially when cyanoacetate derivatives were used as starting materials.

**Scheme 4:**
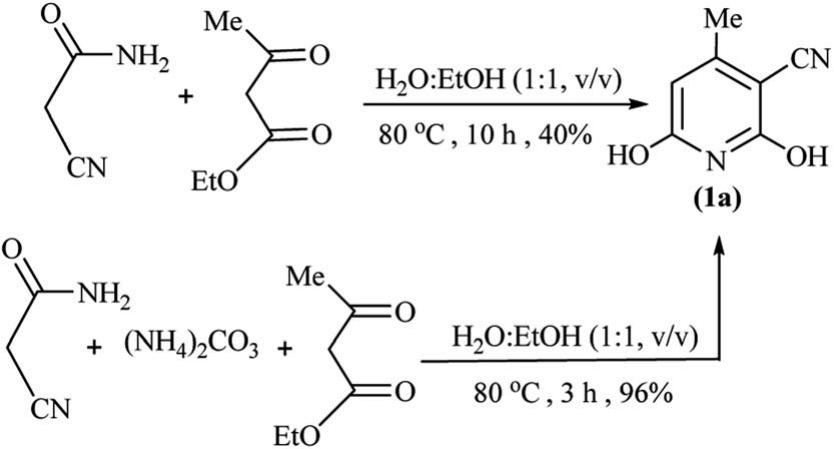
The role of (NH_4_)_2_CO_3_ in GT synthesis of 1a from cyanoacetamide.

To clear the role of (NH_4_)_2_CO_3_ and to support the formation of cyanoacetamide as the reaction intermediate, two parallel reactions of ethyl cyanoacetamide and ethyl acetoacetate in the presence and the absence of ammonium carbonate in water/EtOH solution (1 : 1 volume ratio) were attempted. These reactions gave a 96% and 40% of a significant solitude product after 3 h and 10 h, respectively. Isolation of the product by filtration and NMR analysis showed the formation of product 1a, in both reactions, to reveal the critical role of cyanacetamide as a reaction intermediate. The extension of time from 3 h to 10 h and lowering yield for the reaction run without ammonium carbonate offers the role of (NH_4_)_2_CO_3_ for reaction promotion besides to its role for providing the nitrogen atom of the pyridine ring (Scheme 4).

As often found with MCRs, the simple reaction conditions are compatible with the atmosphere and amenable to up-scaling. Therefore, a 20-fold scale GT reaction of ethyl cyanoacetamide, ethyl acetoacetate, and (NH_4_)_2_CO_3_ was carried out to give the corresponding pyridine product 1a in 95% yield, although reaching the final pH of the reaction media to ∼7 was affected intensively on the reaction yield.

By supporting the formation of cyanoacetamide intermediate in GT reaction, the generality of the advanced GT reaction was tested by condensation of cyanoacetamide and various β-ketoesters or 1,3-diketones at the optimized reaction conditions with (NH_4_)_2_CO_3_ (Table 3).

**Table 3:** (NH_4_)_2_CO_3_-promoted GT reaction of cyanoacetamide with 1,3-dicarbonyls^a^

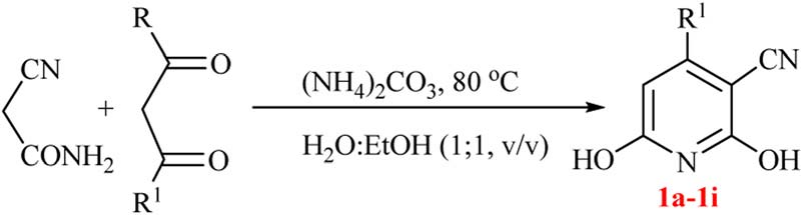					
Entry	R	R^1^	Product	Time (h)	Isolated yield (%)
1	CH_3_	OEt	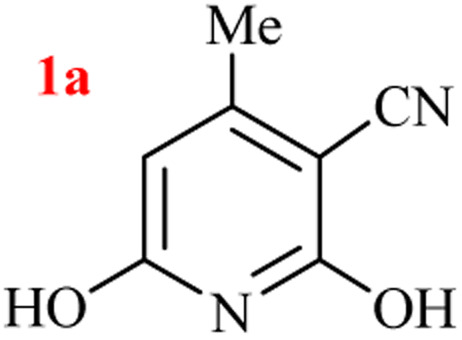	3	97
2	CH_3_	OMe	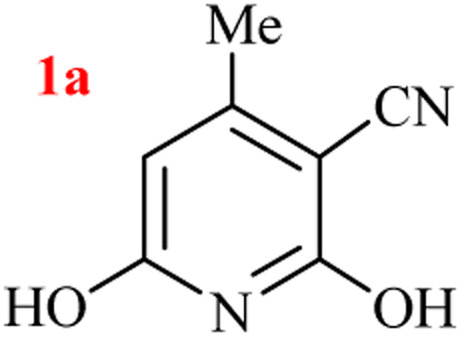	2.5	96
3	CH_3_	O-*t*Bu	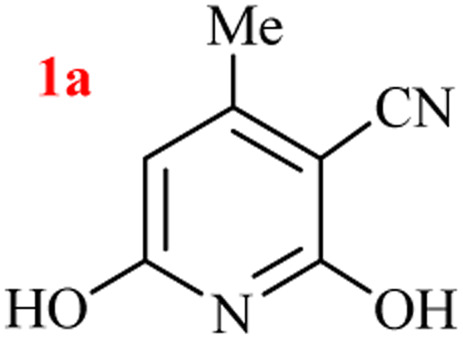	5	91
4	Ph	OEt	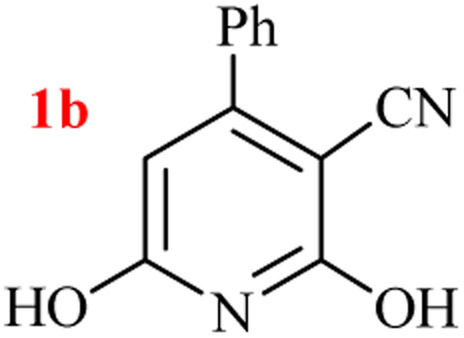	3	95
5	CF_3_	OEt	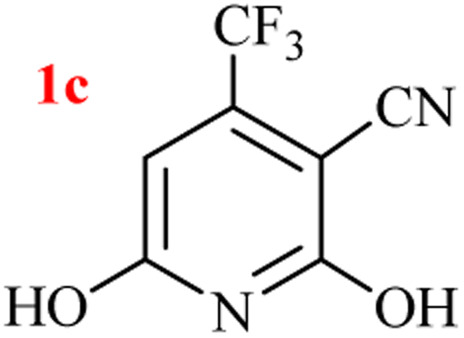	1.5	94
6	CH_2_Cl	OEt	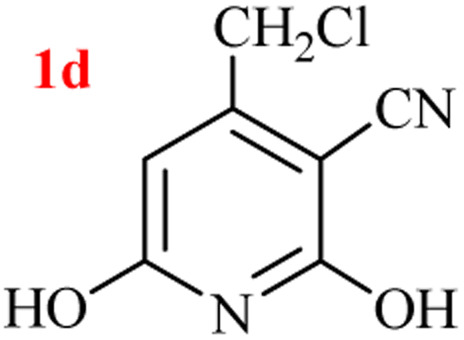	6	78
7	*n*-Propyl	OEt	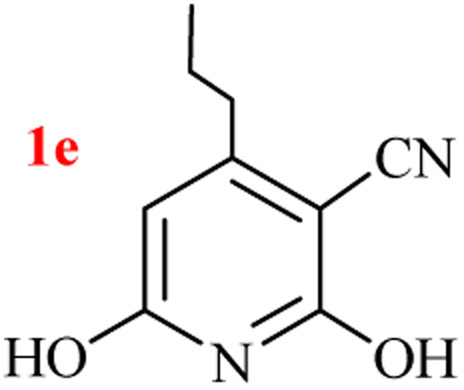	3	80
8	Me	Me	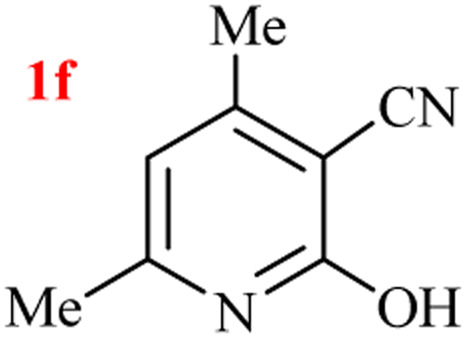	1.5	95
9	Me	Ph	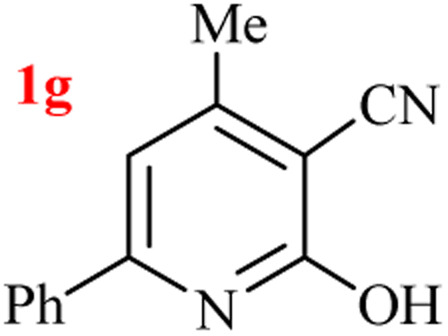	1	95
10	CF_3_	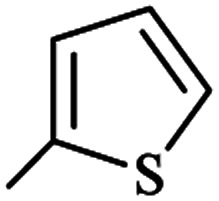	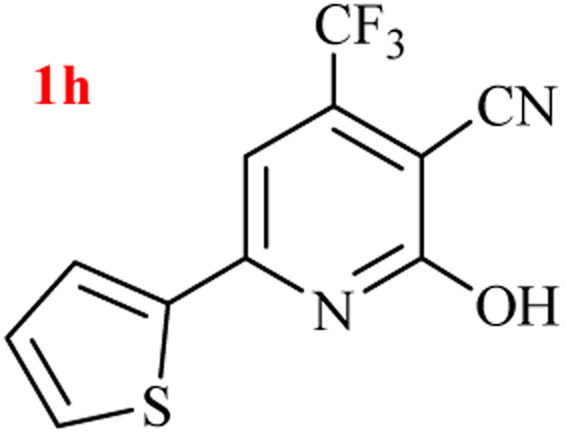	1.5	93
11	CF_3_	Me	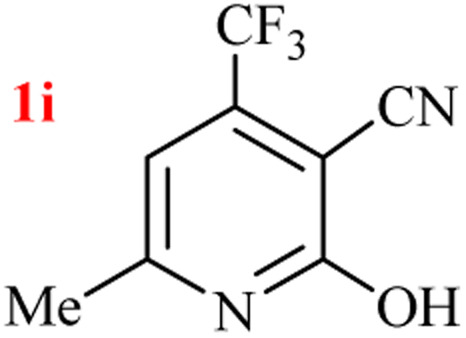	1	97

a Reaction conditions: cyano-acetamide (1 mmol), 1,3-dicarbonyl (1 mmol), (NH_4_)_2_CO_3_ (1 mmol), H_2_O : EtOH (1 : 1, v/v), 80 °C.

**Scheme 5:**
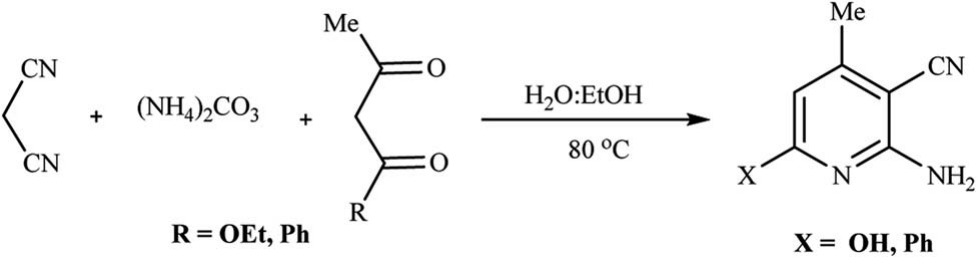
Condensation of malononitrile and ethyl acetoacetate or benzoylacetone with (NH_4_)_2_CO_3_.

Having easy access to such cyano hydroxypyridines, we investigated the three-component condensation of ethyl acetoacetate with malononitrile and ammonium carbonate in the same fashion, which gave the desired 2-amino-3-cyanopyridine product in excellent yields after the simple addition of water. By running a similar experiment with benzoylacetone, the reaction proceeded smoothly to give the corresponding product in high yield (Scheme 5) (see also the ESI† for details).

## 3 Conclusion

In conclusion, we have developed an advanced version of the Guareschi–Thorpe reaction in an aqueous medium of ammonium carbonate as either a solid ammonia source or buffered catalyst for the high yielding synthesis of a variety of known and new hydroxy cyanopyridines from either alkyl cyanoesters or cyanoacetamide and 1,3-dicarbonyls. The efficiency of this protocol is based on the accessibility of starting materials, high yields and purities of products, simple work-up without the use of organic solvents, and eco-environmentally friendly green conditions.

## 4 Experimental

### 4.1 General

All chemicals were purchased from Sigma-Aldrich and Merck (Germany) in analytical grade and used without further purification. Reactions followed by thin layer chromatography on silica gel 60 F_254_ (Merck). Fourier transform infrared (FT-IR) spectra were recorded on a Bruker Equinox 55 FT-IR spectrometer in the range of 4000–400 cm^−1^ using KBr discs. Melting points were determined with a Buchi B-540 apparatus and are uncorrected. NMR spectra of products were recorded as a DMSO-d_6_ solution in a Bruker 500 MHz spectrophotometer instrument, chemical shifts and *J*-values are in ppm and Hz, respectively.

### 4.2 General procedure for the three-component GT synthesis of hydroxyl-cyano-pyridines

A mixture of a 1,3-dicarbonyl compound (1 mmol), alkyl cyanoacetate (1 mmol), and ammonium carbonate (2 mmol) in EtOH (1 mL) : H_2_O (1 mL) mixture was stirred at 80 °C for the given time. Solidification of the final products took place during this period. After the reaction completion (TLC monitoring), cold water was added, the precipitated solid product was filtered, washed with water, dried, and characterized with no further purification by melting point, FT-IR, or NMR spectra (see ESI S_3_–S_26_†).

### 4.3 General procedure for the two-component synthesis of hydroxyl-cyano-pyridines

A mixture of a 1,3-dicarbonyl compound (1 mmol), cyanoacetamide (1 mmol), and ammonium carbonate (1 mmol) in EtOH (1 mL) : H_2_O (1 mL) mixture was stirred at 80 °C for the given time. And the solid product was isolated and characterized as above.

## Conflicts of interest

The authors declare no competing interest.

## Supplementary Material

RA-013-D3RA04590K-s001

## References

[cit1] Peloquin D. M., Schmedake T. A. (2016). Coord. Chem. Rev..

[cit2] Liu J., Ding Q., Fang W., Wu W., Zhang Y., Peng Y. (2018). J. Org. Chem..

[cit3] Soldatenkov A. T. , Pozharskii A. F. and Katritzky A. R., Heterocycles in Life and Society: An Introduction to Heterocyclic Chemistry, Biochemistry and Applications, John Wiley & Sons, 2011

[cit4] Mermer A., Keles T., Sirin Y. (2021). Bioorg. Chem..

[cit5] Lacerda R. B., de Lima C. K., da Silva L. L., Romeiro N. C., Miranda A. L. P., Barreiro E. J., Fraga C. A. (2009). Bioorg. Med. Chem..

[cit6] Torabi M. , Yarie M., Baghery S. and Zolfigol M. A., Recent Developments in the Synthesis and Applications of Pyridines, 2023, pp. 503–580

[cit7] Arlan F. M., Marjani A. P., Javahershenas R., Khalafy J. (2021). New J. Chem..

[cit8] Li J. J. and Li J. J., Name Reactions: A Collection of Detailed Reaction Mechanisms, 2003, pp. 168–168

[cit9] Jaiswal P. K., Sharma V., Mathur M., Chaudhary S. (2018). Org. Lett..

[cit10] Eriksson M. C., Zeng X., Xu J., Reeves D. C., Busacca C. A., Farina V., Senanayake C. H. (2018). Synlett.

[cit11] Jin X., Xing L., Deng D. D., Yan J., Fu Y., Dong W. (2022). J. Org. Chem..

[cit12] Hurtado-Rodríguez D., Salinas-Torres A., Rojas H., Becerra D., Castillo J.-C. (2022). RSC Adv..

[cit13] Bensaude O., Chevrier M., Dubois J. (1978). J. Am. Chem. Soc..

[cit14] Bensaude O., Chevrier M., Dubois J. E. (1979). J. Am. Chem. Soc..

[cit15] Mohammad Abu-Taweel G., Ibrahim M. M., Khan S., Al-Saidi H. M., Alshamrani M., Alhumaydhi F. A., Alharthi S. S. (2022). Crit. Rev. Anal. Chem..

[cit16] De S., SK A. K., Shah S. K., Kazi S., Sarkar N., Banerjee S., Dey S. (2022). RSC Adv..

[cit17] Aimo A., Cerbai E., Bartolucci G., Adamo L., Barison A., Surdo G. L., Biagini S., Passino C., Emdin M. (2020). Pharmacol. Res..

[cit18] Kang J.-A., Kim S., Park M., Park H.-J., Kim J.-H., Park S., Hwang J.-R., Kim Y.-C., Jun Kim Y., Cho Y. (2019). Nat. Commun..

[cit19] Friedli M. J., Inestrosa N. C. (2021). Molecules.

[cit20] Hantzsch A. (1881). Ber. Dtsch. Chem. Ges..

[cit21] Kon G. A. R., Thorpe J. F. (1919). J. Chem. Soc., Trans..

[cit22] Collins D. J., James A. M. (1989). Aust. J. Chem..

[cit23] Carles L., Narkunan K., Penlou S., Rousset L., Bouchu D., Ciufolini M. A. (2002). J. Org. Chem..

[cit24] Hessel V., Tran N. N., Asrami M. R., Tran Q. D., Long N. V. D., Escribà-Gelonch M., Tejada J. O., Linke S., Sundmacher K. (2022). Green Chem..

[cit25] Zhou F., Hearne Z., Li C.-J. (2019). Curr. Opin. Green Sustainable Chem..

[cit26] Cortes-Clerget M., Yu J., Kincaid J. R., Walde P., Gallou F., Lipshutz B. H. (2021). Chem. Sci..

[cit27] Lipshutz B. H., Ghorai S., Cortes-Clerget M. (2018). Chem. – Eur. J..

[cit28] Gan L., Wei L., Wan J. P. (2020). ChemistrySelect.

[cit29] Wu X., Larhed M. (2005). Org. Lett..

[cit30] Kokel A., Schäfer C., Török B. (2017). Green Chem..

[cit31] Auria-Luna F., Fernández-Moreira V., Marqués-López E., Gimeno M. C., Herrera R. P. (2020). Sci. Rep..

[cit32] Kitanosono T., Masuda K., Xu P., Kobayashi S. (2018). Chem. Rev..

[cit33] Rahimi Z., Bayat M., Hosseini H. (2022). RSC Adv..

[cit34] Cioc R. C., Ruijter E., Orru R. V. (2014). Green Chem..

[cit35] Javanbakht S., Nasiriani T., Farhid H., Nazeri M. T., Shaabani A. (2022). Front. Chem. Sci. Eng..

[cit36] Mohammadi P., Sheibani H. (2019). Mater. Chem. Phys..

[cit37] Vachan B., Karuppasamy M., Vinoth P., Vivek Kumar S., Perumal S., Sridharan V., Menéndez J. C. (2020). Adv. Synth. Catal..

[cit38] Tamaddon F., Arab D., Ahmadi-AhmadAbadi E. (2020). Carbohydr. Polym..

[cit39] Tamaddon F., Khorram A. (2020). J. Mol. Liq..

[cit40] Tamaddon F., Arab D. (2019). Int. J. Biol. Macromol..

[cit41] Tamaddon F., Ghazi S. (2015). Catal. Commun..

[cit42] Tamaddon F., Moradi S. (2013). J. Mol. Catal. A: Chem..

[cit43] Tamaddon F., Razmi Z., Jafari A. A. (2010). Tetrahedron Lett..

[cit44] Tamaddon F., Azadi D. (2018). J. Mol. Liq..

