# Supplementary material for: Advanced Guareschi–Thorpe synthesis of pyridines in green buffer, and pH-controlled aqueous medium with ammonium carbonate
Source: RSC Adv. 2023 Aug 21;13(35):24846–53. doi: 10.1039/d3ra04590k (PMC10440631; doi:10.1039/d3ra04590k)
Supplement: RA-013-D3RA04590K-s001 [file RA-013-D3RA04590K-s001.pdf]

Electronic Supplementary Material (ESI) for RSC Advances.
This journal is © The Royal Society of Chemistry 2023

Supporting Information

Advanced and green Guareschi-Thorpe synthesis of pyridines at bufferic pH-controlled aqueous medium with ammonium carbonate

Fatemeh Tamaddon[a], Sajedeh Maddah-Roodan

[a] Department of Chemistry, Yazd University, Yazd 89195-741, Iran

Ph.D. student in organic chemistry, Department of Chemistry, Yazd University, Yazd 89195-741, Iran

E-mail: ftamaddon@yazd.ac.ir or sajedeh.maddah@stu.yazd.ac.ir

List of contents

Preparation of cyanopyridines with ethyl cyanoacetate and β-ketoesters or 1,3-diketones: S3
Preparation of cyanopyridines with cyanoacetamide and β-ketoesters or 1,3-diketones: S3
Scope of cyanopyridines.........S4
Analytical and spectra data for selected products.........S4
FT-IR (KBr) spectrum of (**1a**).........S6
^{1}H NMR (500 MHz, DMSO-d_6) spectrum of (**1a**).........S7
Expand of ^{1}H NMR (500 MHz, DMSO-d_6) spectrum of (**1a**).........S7
^{13}C NMR (125 MHz, DMSO-d_6) spectrum of (**1a**).........S8
FT-IR (KBr) spectrum of (**1c**).........S9
FT-IR (KBr) spectrum of (**1e**).........S10
^{1}H NMR (500 MHz, DMSO-d_6) spectrum of (**1e**).........S11
Expand of ^{1}H NMR (500 MHz, DMSO-d_6) spectrum of (**1e**).........S11
^{13}C NMR (125 MHz, DMSO-d_6) spectrum of (**1e**).........S12
FT-IR (KBr) spectrum of (**1f**).........S13
^{1}H NMR (500 MHz, DMSO-d_6) spectrum of (**1f**).........S14
^{13}C NMR (125 MHz, DMSO-d_6) spectrum of (**1f**).........S15
FT-IR (KBr) spectrum of (**1g**).........S16
^{1}H NMR (500 MHz, DMSO-d_6) spectrum of (**1g**).........S17
Expand of ^{1}H NMR (500 MHz, DMSO-d_6) spectrum of (**1g**).........S17

FT-IR (KBr) spectrum of (**1h**)....S18
^{1}H NMR (500 MHz, DMSO-d_6) spectrum of (**1h**)....S19
FT-IR (KBr) spectrum of (**1i**)....S20
^{1}H NMR (500 MHz, DMSO-d_6) spectrum of (**1i**)....S21
Expand of ^{1}H NMR (500 MHz, DMSO-d_6) spectrum of (**1i**)....S21
^{13}C NMR (125 MHz, DMSO-d_6) spectrum of (**1i**)....S22
FT-IR (KBr) spectrum of (**2a**)....S23
FT-IR (KBr) spectrum of (**2b**)....S24
^{1}H NMR (500 MHz, DMSO-d_6) spectrum of (**2b**)....S25
Expand of ^{1}H NMR (500 MHz, DMSO-d_6) spectrum of (**2b**)....S25
^{13}C NMR (125 MHz, DMSO-d_6) spectrum of (**2b**)....S26
Expand of ^{13}C NMR (125 MHz, DMSO-d_6) spectrum of (**2b**)....S26

Preparation of cyanopyridines with ethyl cyanoacetate and β-ketoesters or 1,3-diketones:

A mixture of β-ketoester or 1,3-diketone (1 mmol), ethyl cyanoacetate (1 mmol, 0.11 mL) and $(NH_4)_2CO_3$ (2 mmol, 0.192 g) in H_2O and EtOH (1:1, v/v) was stirred vigorously at 80 °C for 5-8 h in a round-bottom flask. The progress of the reaction was monitored *via* TLC (EtOAc: Hexane) (30:70) analysis. After the reaction finishing, the precipitated product by adding ice cold water (20 mL) to the reaction mixture was filtered, washed, and dried..

Preparation of cyanopyridines with cyanoacetamide and β-ketoesters or 1,3-diketones:

A solution containing cyanoacetamide (1 mmol), ethyl cyanoacetate (1 mmol, 0.11 mL) and $(NH_4)_2CO_3$ (1 mmol, 0.096 g) in 2 mL of H_2O and EtOH (1:1) was stirred at 80 °C for 1-6 h. After completion of the reaction, the resulting solid was collected like the previous method.

Scope of cyanopyridines:

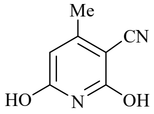

(1a)

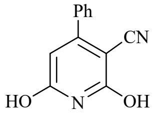

(1b)

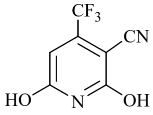

(1c)

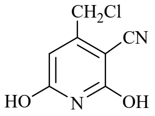

(1d)

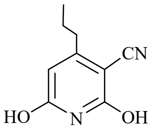

(1e)

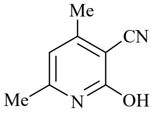

(1f)

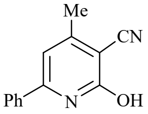

(1g)

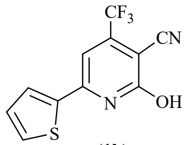

(1h)

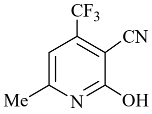

(1i)

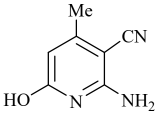

(2a)

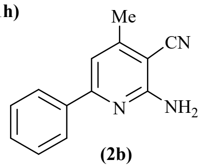

(2b)

Analytical and spectra data for selected products:

2,6-Dihydroxy-4-methylpyridine-3-carbonitrile **(1a)**

white solid, m.p.: 323-328, FT-IR (KBr): υ_{max} 3150-2900 (OH stretching), 2223 (C≡N), 1598 (C=N) cm^{-1}. ^{1}H NMR (500 MHz, DMSO-d_6): δ_H 2.22 (s, 3H, CH_3), 5.59 (s, 1H, CH ring), 12.00 (br s, 2H, OH) ppm. ^{13}C NMR (125 MHz, DMSO-d_6): δ_c 21.5, 90.1, 93.5, 117.9, 161.2, 161.8, 162.7 ppm.

4-(Trifluoromethyl)-2,6-dihydroxypyridine-3-carbonitrile **(1c)**

white solid, m.p.; 225-230 °C, FT-IR (KBr): υ_{max} 3391-2934 (OH stretching), 2226 (C≡N), 1618 (C=N ring) cm^{-1}.

2,6-Dihydroxy-4-propylpyridine-3-carbonitrile **(1e)**

white solid, m.p.: 295-300 °C, FT-IR (KBr): υ_{max} 3200-2800 (OH stretching), 2223 (C≡N), 1599 (C=N ring) cm^{-1}. ^{1}H NMR (500 MHz, DMSO-d_6): δ_H 0.92 (t, *J*=7 Hz, 3H, CH_3), 1.57 (m, *J*=7.5 Hz, 2H, $\underline{CH_2}CH_3$), 2.49 (t, *J*=7.5 Hz, 2H, $\underline{CH_2}CH_2$), 4.90 (br s, 2H, OH) 5.58 (s, 1H, CH ring) ppm. ^{13}C NMR (125 MHz, DMSO-d_6): δ_c 14.2, 14.6, 21.8, 32.2, 46.5, 59.9, 61.2, 80.0, 109.9, 111.4, 111.5, 115.0, 145.2, 149.5, 165.9, 168.9 ppm.

2-Hydroxy-4,6-dimethylpyridine-3-carbonitrile **(1f)**

white solid, m.p.: 367-370 °C, FT-IR (KBr): υ_{max} 3100-2800 (OH stretching), 2218 (C≡N), 1618 (C=N ring) cm^{-1}. ^{1}H NMR (500 MHz, DMSO-d_6): δ_H 2.21 (s, 3H, CH_3), 2.33 (s, 3H, CH_3), 6.13 (s, 1H, CH ring), 12.24 (s, 1H, OH) ppm. ^{13}C NMR (125 MHz, DMSO-d_6): δ_c 19.7, 21.5, 99.9, 108.3, 116.6, 152.1, 161.3, 161.4 ppm.

2-Hydroxy-4-methyl-6-phenylpyridine-3-carbonitrile **(1g)**

white solid, m.p.: 290-296, FT-IR (KBr): υ_{max} 3100-3000 (OH stretching), 2222 (C≡N), 1608 (C=N ring) cm^{-1}. ^{1}H NMR (500 MHz, DMSO-d_6): δ_H 2 (s, 3H, $_3$), 5.70 (s, 1H), 7.83 (d, *J*=7 Hz, 2H), 7.41-7.45 (m, *J*=7 Hz, 3H), 10.03 (s, 1H, OH) ppm.

4-(Trifluoromethyl)-2-hydroxy-6-(thiophen-2-yl) pyridine-3-carbonitrile **(1h)**

yellow solid, m.p.: 285 °C, FT-IR (KBr): ῡ$_{max}$ 3102 (NH stretching), 2231 (C≡N), 1642 (C=O) cm^{-1}. ^{1}H NMR (500 MHz, DMSO-d_6): δ_H 7.27 (t, *J*=4 Hz, 1H, CH ring), 7.67 (s, 1H, CH ring), 7.91 (d, *J*=5 Hz, 1H, CH ring), 8.19 (s, 1H, CH ring), 13-14 (br s, 1H, OH) ppm.

4-(Trifluoromethyl)-2-hydroxy-6-methylpyridine-3-carbonitrile **(1i)**
white solid. M.p.: 232-236 °C, FT-IR (KBr): ῡ$_{max}$ 3102 (NH stretching), 2231 (C≡N), 1641 (C=O) cm^{-1}. ^{1}H NMR (500 MHz, DMSO-d_6): δ_H 2.38 (s, 3H, CH_3), 6.61 (s, 1H, CH ring), 13.35 (s, 1H, OH) ppm. ^{13}C NMR (125 MHz, DMSO-d_6): δ_c 19.5, 96.1, 101.5, 113.4, 121.9 (q, *J*=273.75 Hz, CF_3), 145.8 (q, *J*=32.5 Hz, C-CF_3), 156.9, 160.4 ppm.

2-Amino-4-methyl-6-hydroxypyridine-3-carbonitrile (**2a**)
pale yellow solid, m.p.: 132-134 °C FT-IR (KBr): ῡ$_{max}$ 3381, 3230, 3326 (NH stretching), 2215 (C≡N), 1723 (C=O), 1646 (C=N ring) cm^{-1}.

2-Amino-4-methyl-6-phenylpyridine-3-carbonitrile (**2b**)
pale pink solid, m.p.: 185-189 °C FT-IR (KBr): ῡ$_{max}$ 3469, 3344, 3239 (NH stretching), 2211 (C≡N), 1640 (C=N ring) cm^{-1}. ^{1}H NMR (500 MHz, DMSO-d_6): δ_H 2.45 (s, 3H, CH_3), 6.68 (s, 2H, NH), 6.75 (s, 1H, CH ring), 7.51-7.54 (m, 5H) ppm.^{13}C NMR (125 MHz, DMSO-d_6): δ_c 21.4, 115.9, 116.6, 119.5, 128.8, 129.1, 129.7, 148.7, 150.1, 153.9 ppm.

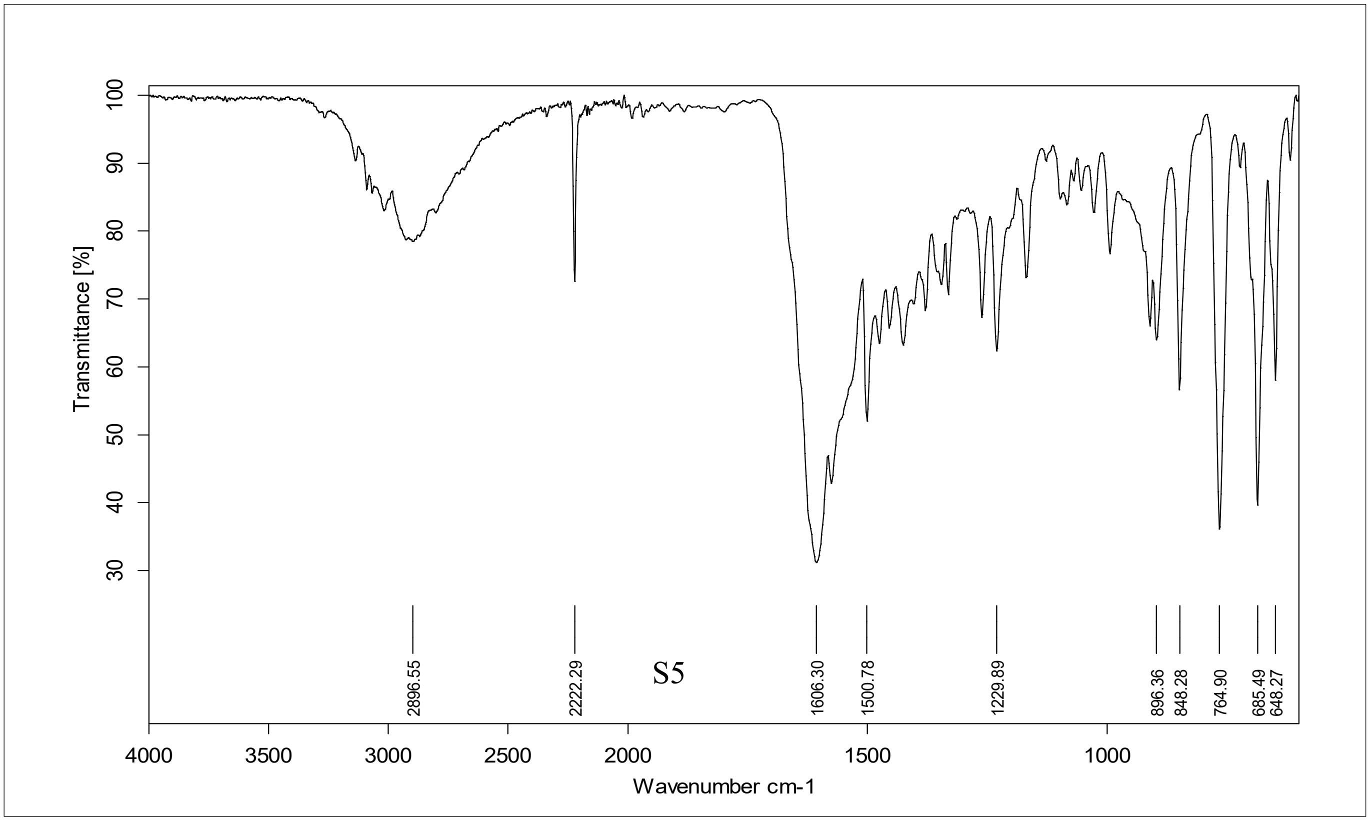

FT-IR (KBr) spectrum of (**1a**)

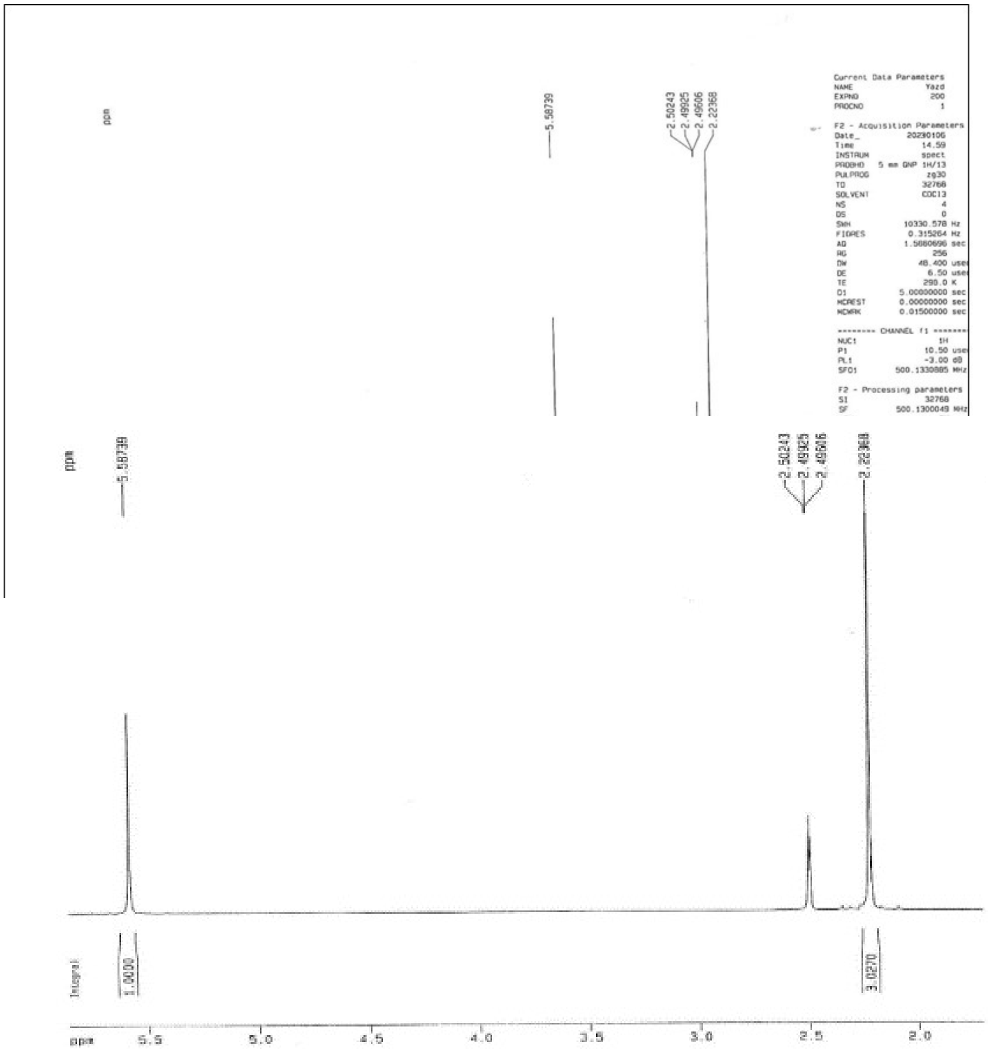

Expand of ^{1}H NMR (500 MHz, DMSO-d_6) spectrum of (**1a**)

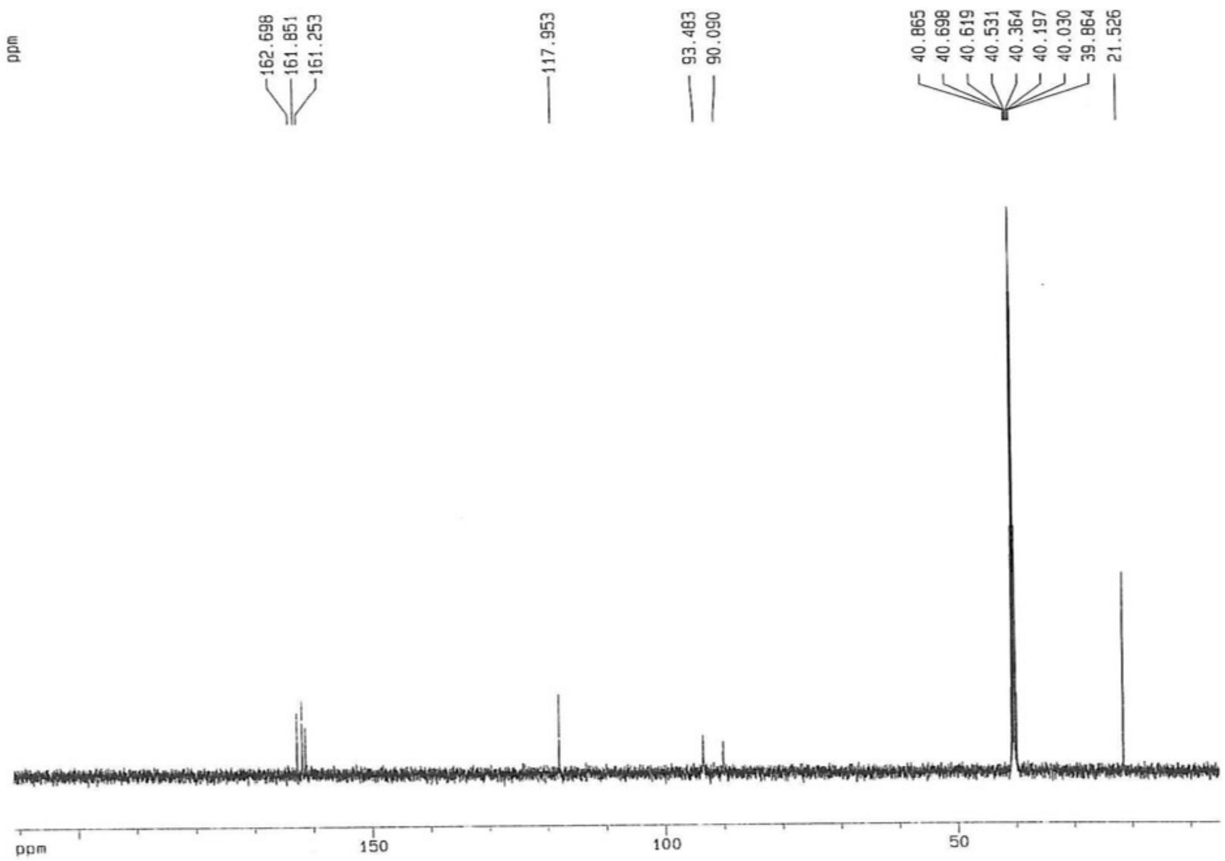

^{13}C NMR (125 MHz, DMSO-d_6) spectrum of (**1a**)

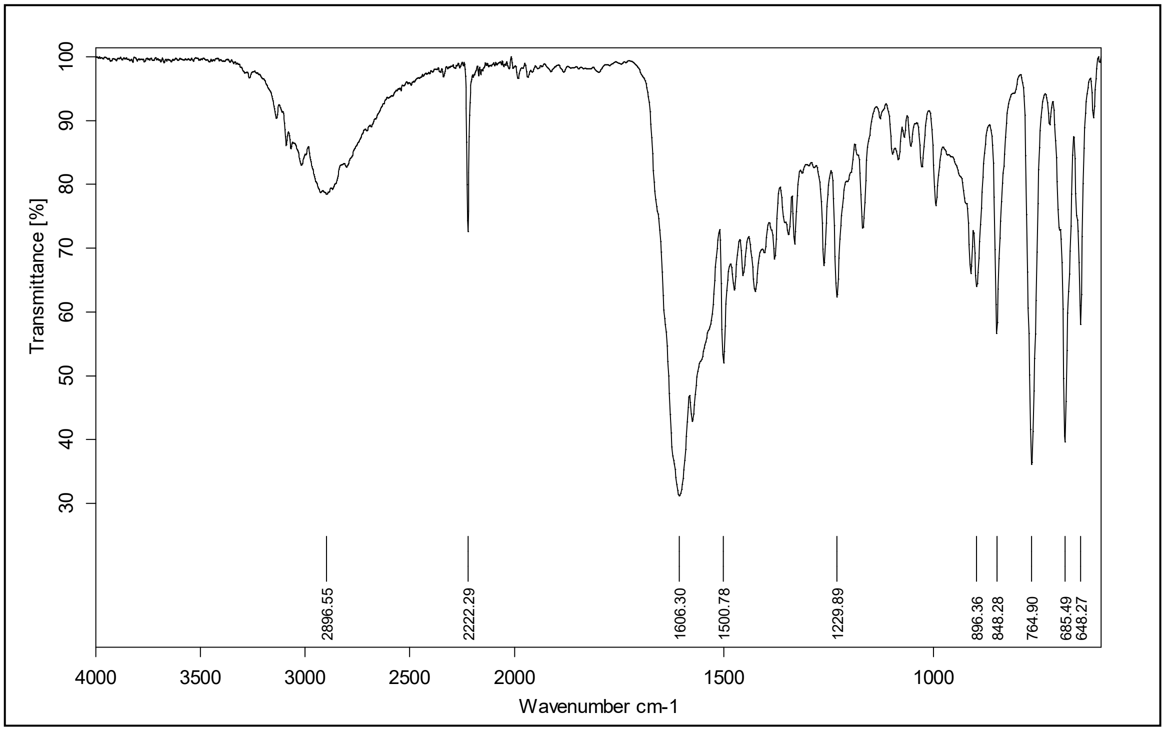

FT-IR (KBr) spectrum of (**1c**)

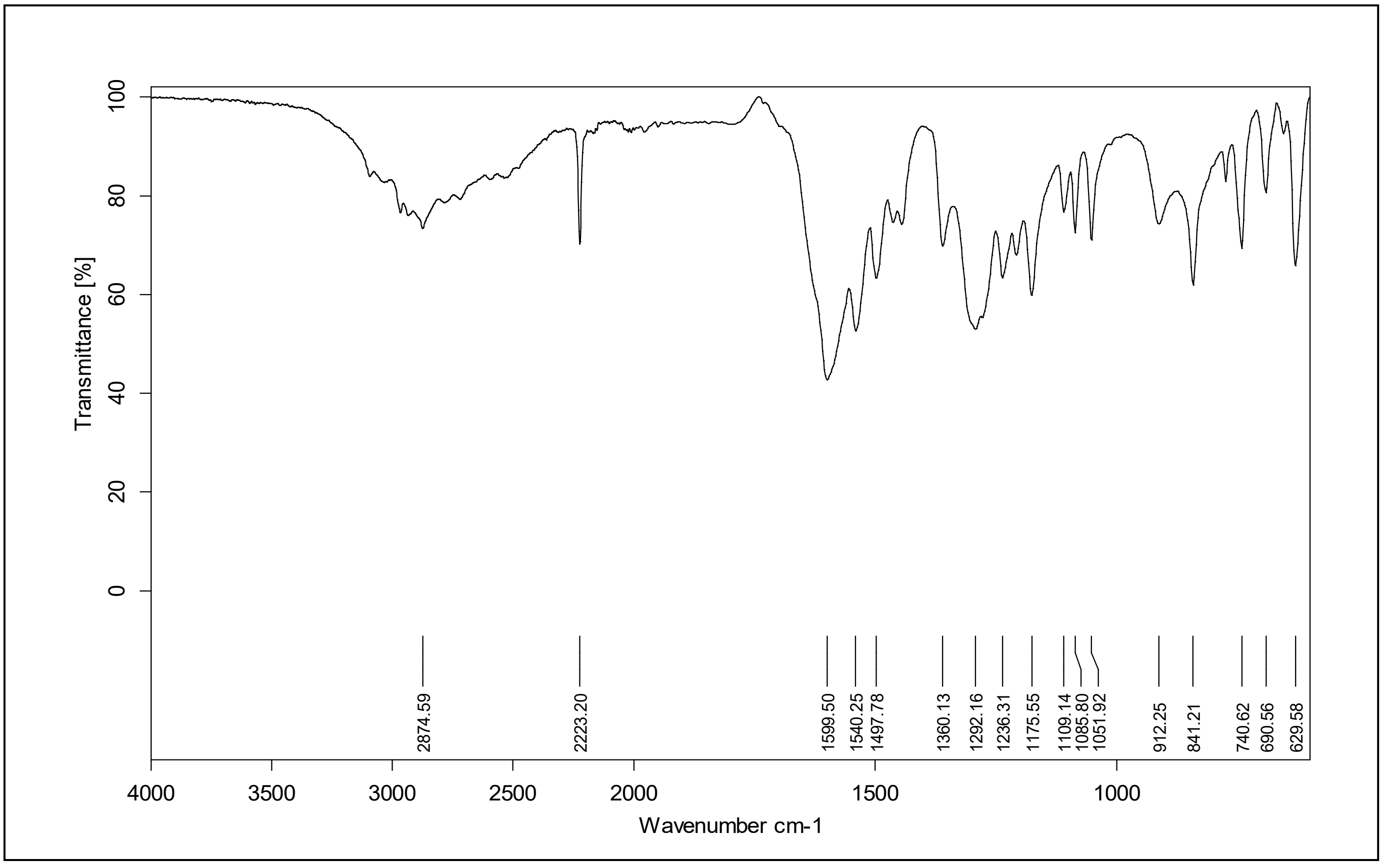

FT-IR (KBr) spectrum of (**1e**)

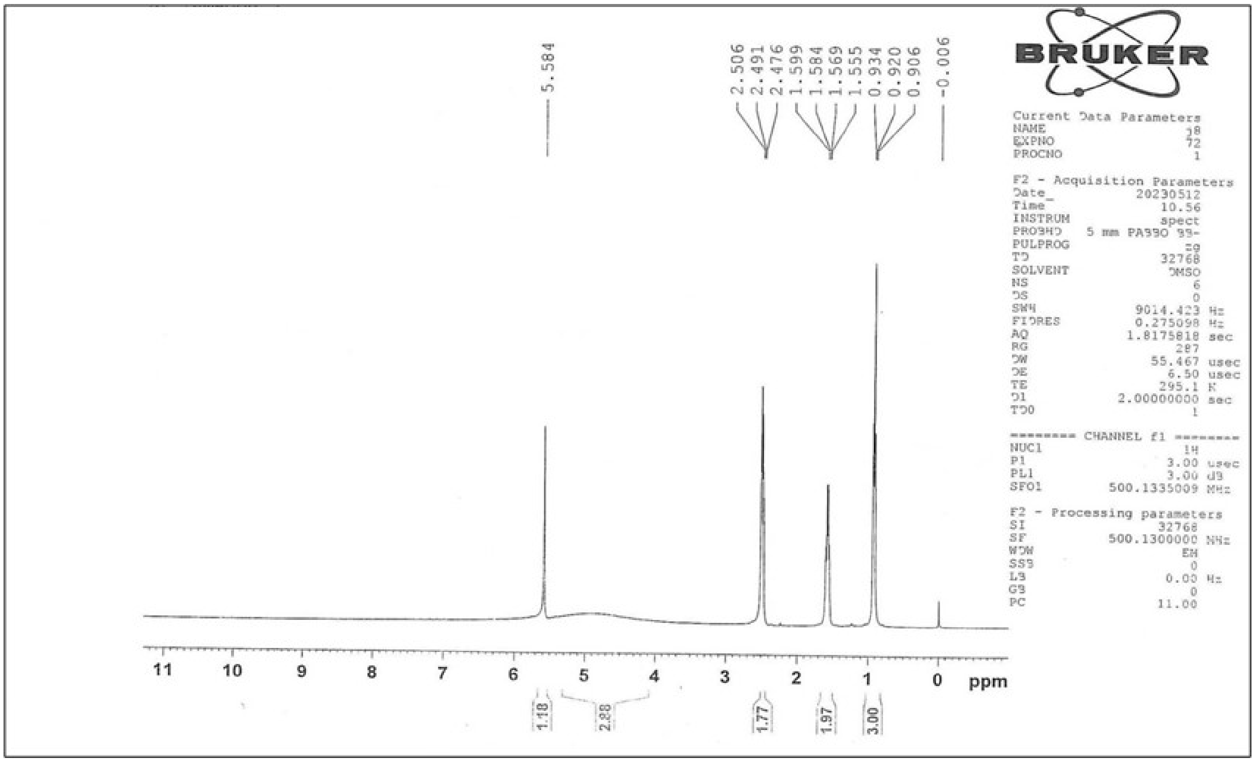

^1H NMR (500 MHz, DMSO-d_6) spectrum of (**1e**)

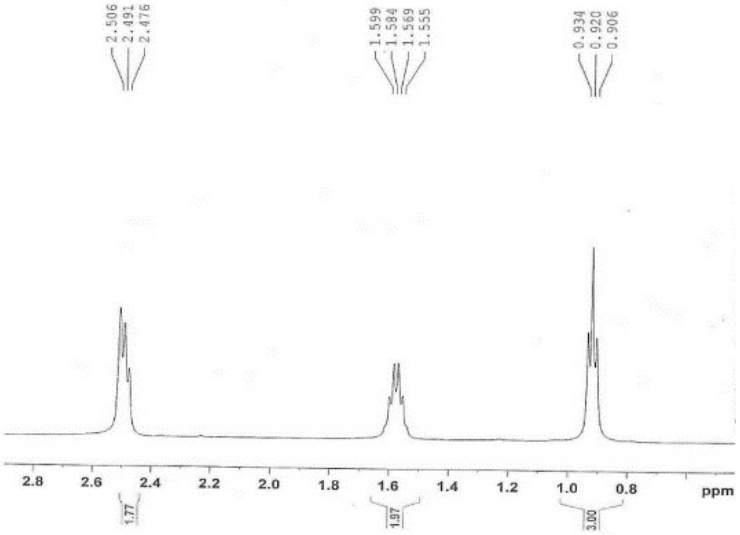

Expand of ^1H NMR (500 MHz, DMSO-d_6) spectrum of (**1e**)

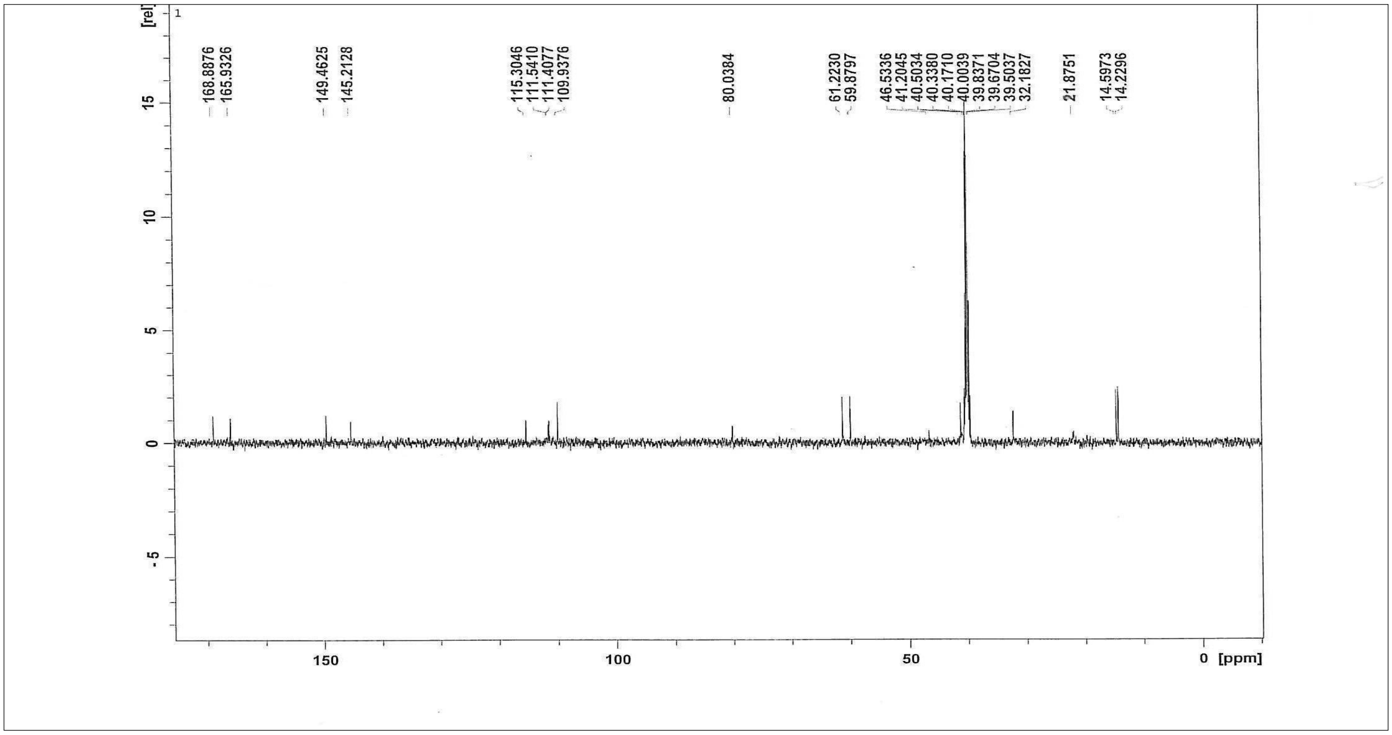

^{13}C NMR (125 MHz, DMSO-d_6) spectrum of (**1e**)

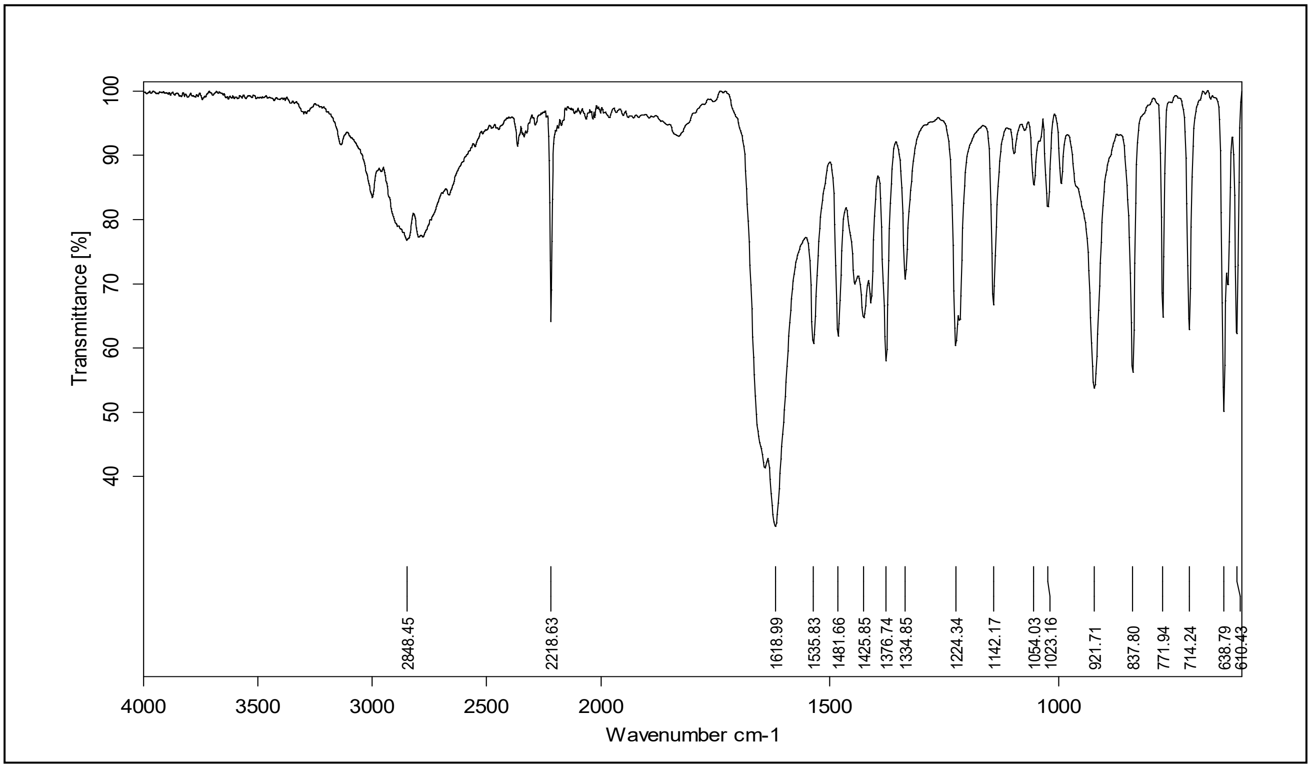

FT-IR (KBr) spectrum of (**1f**)

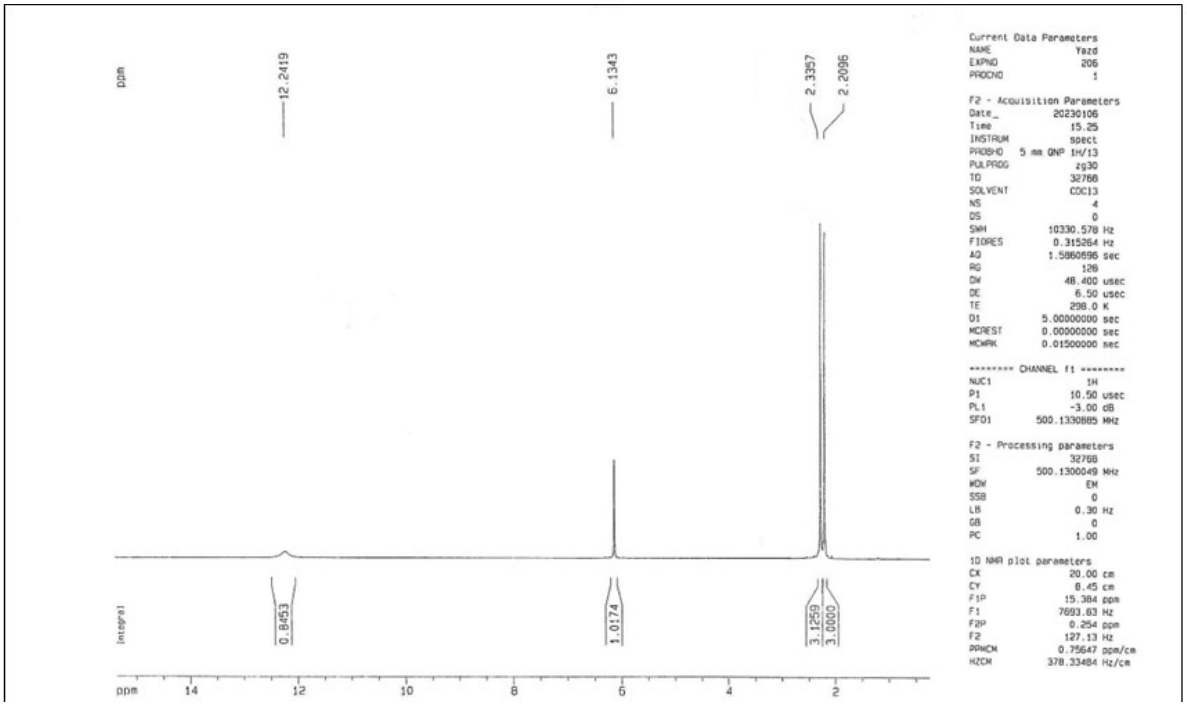

^{1}H NMR (500 MHz, DMSO-d_6) spectrum of (**1f**)

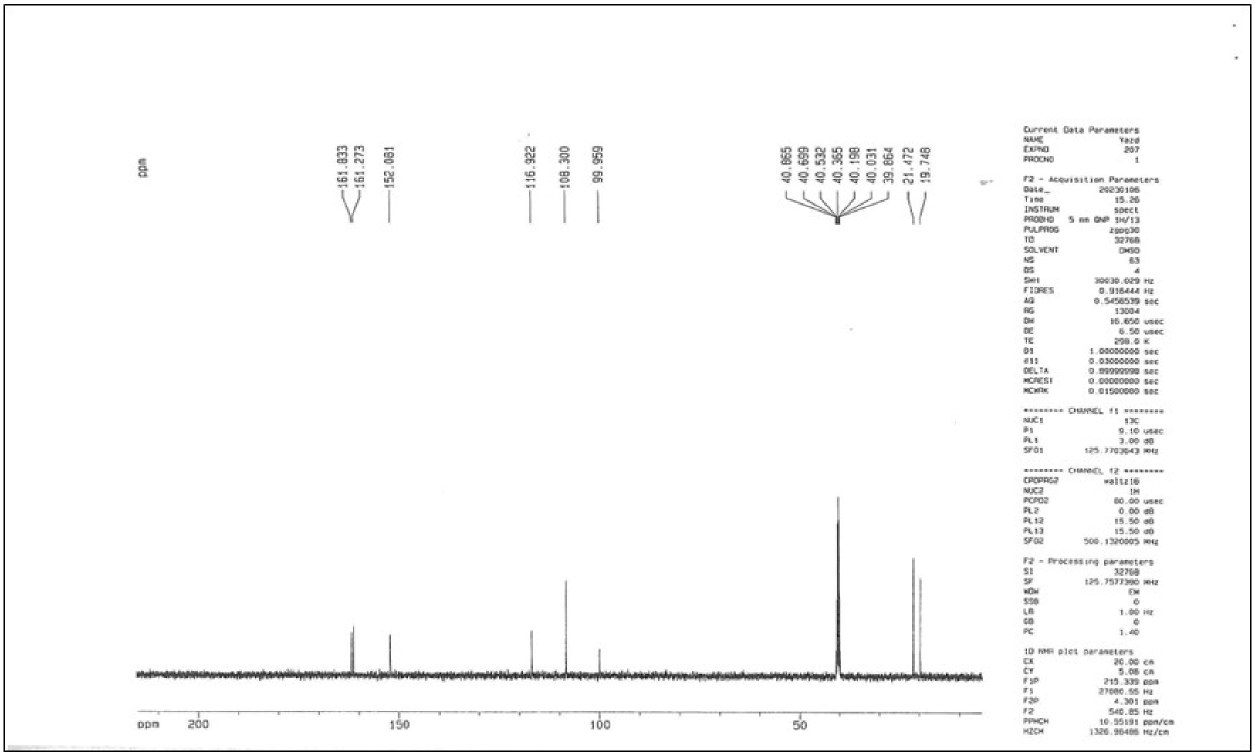

^{13}C NMR (125 MHz, DMSO-d_6) spectrum of (**1f**)

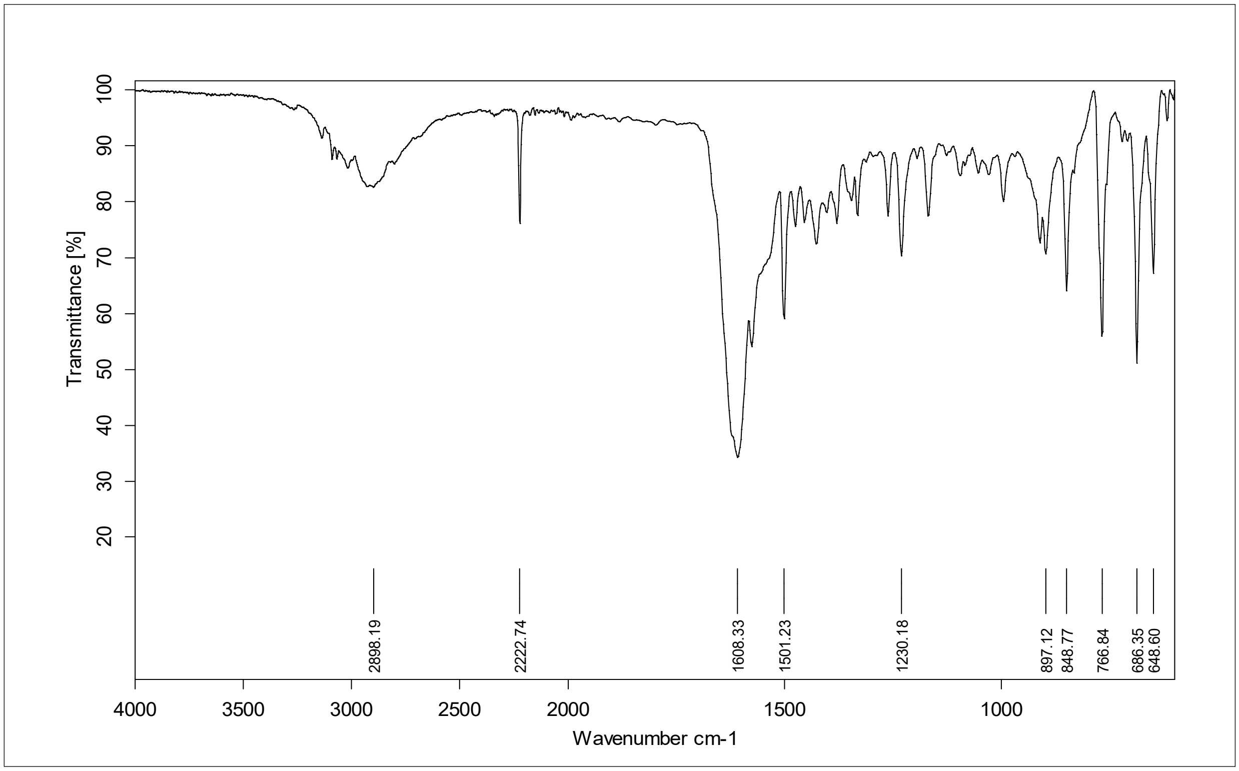

FT-IR (KBr) spectrum of (**1g**)

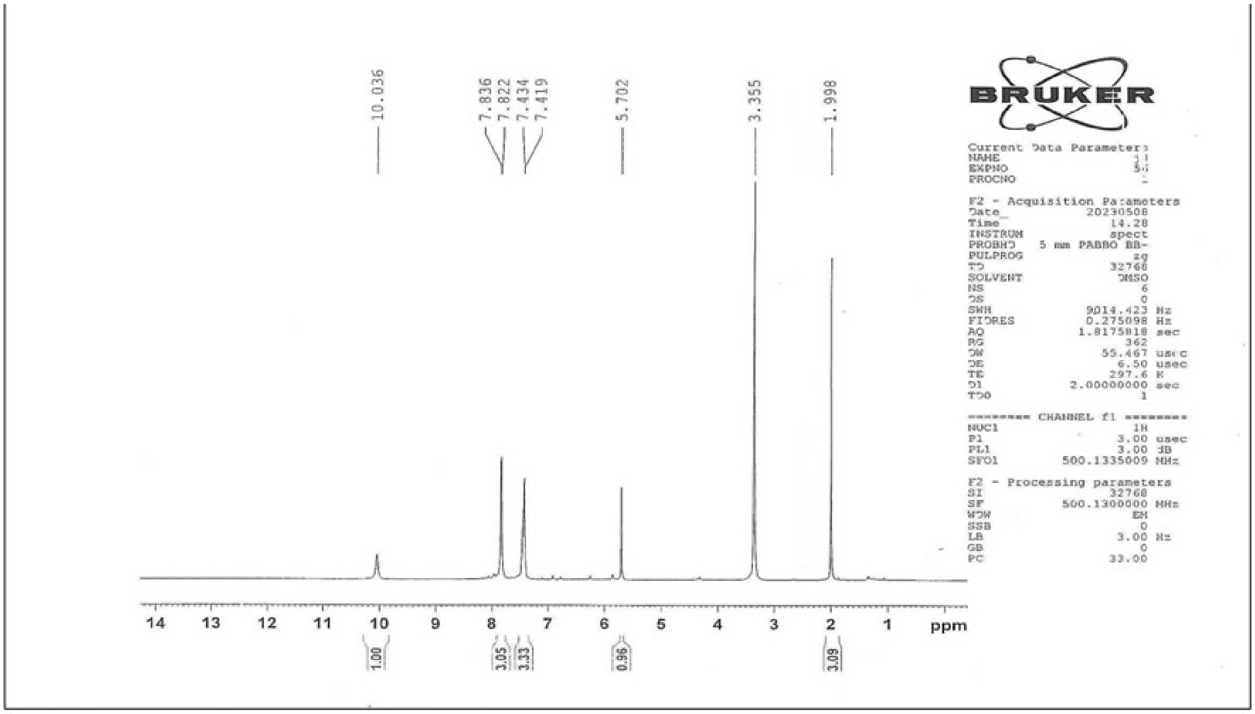

^{1}H NMR (500 MHz, DMSO-d_6) spectrum of (**1g**)

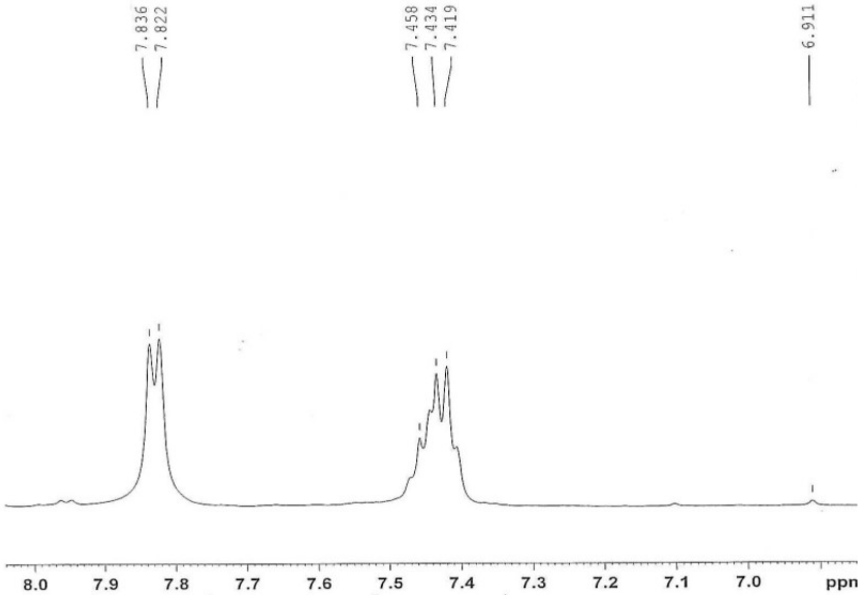

Expand of ^{1}H NMR (500 MHz, DMSO-d_6) spectrum of (**1g**)

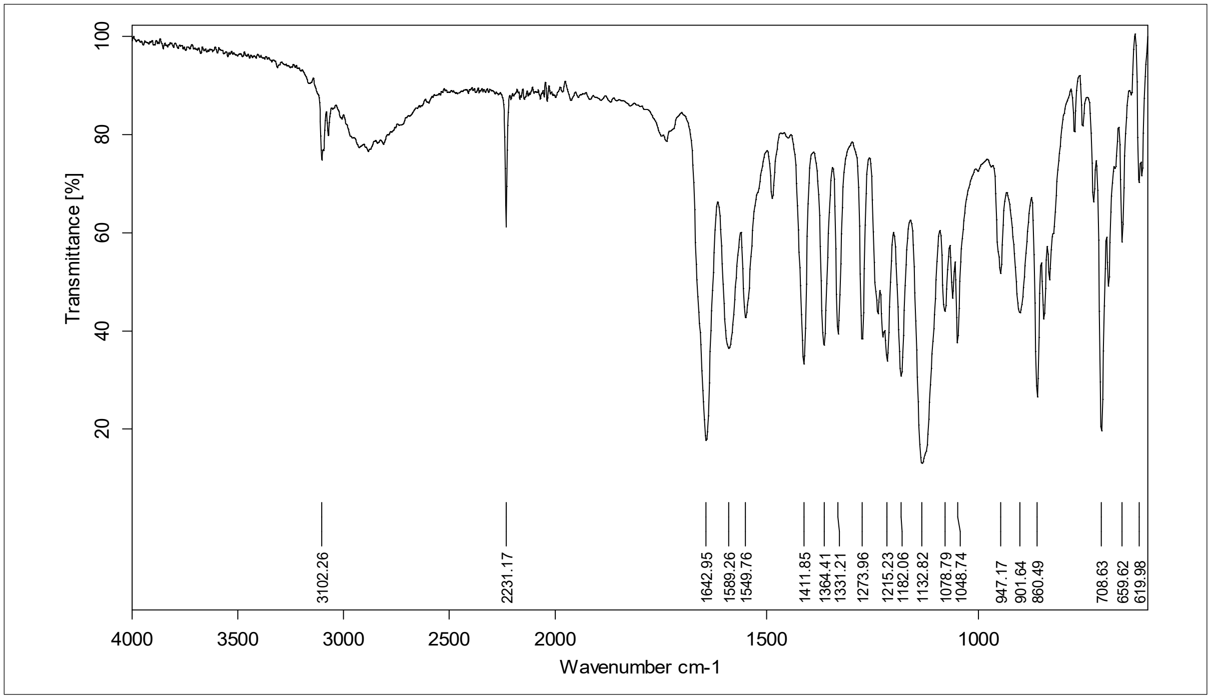

FT-IR (KBr) spectrum of (**1h**)

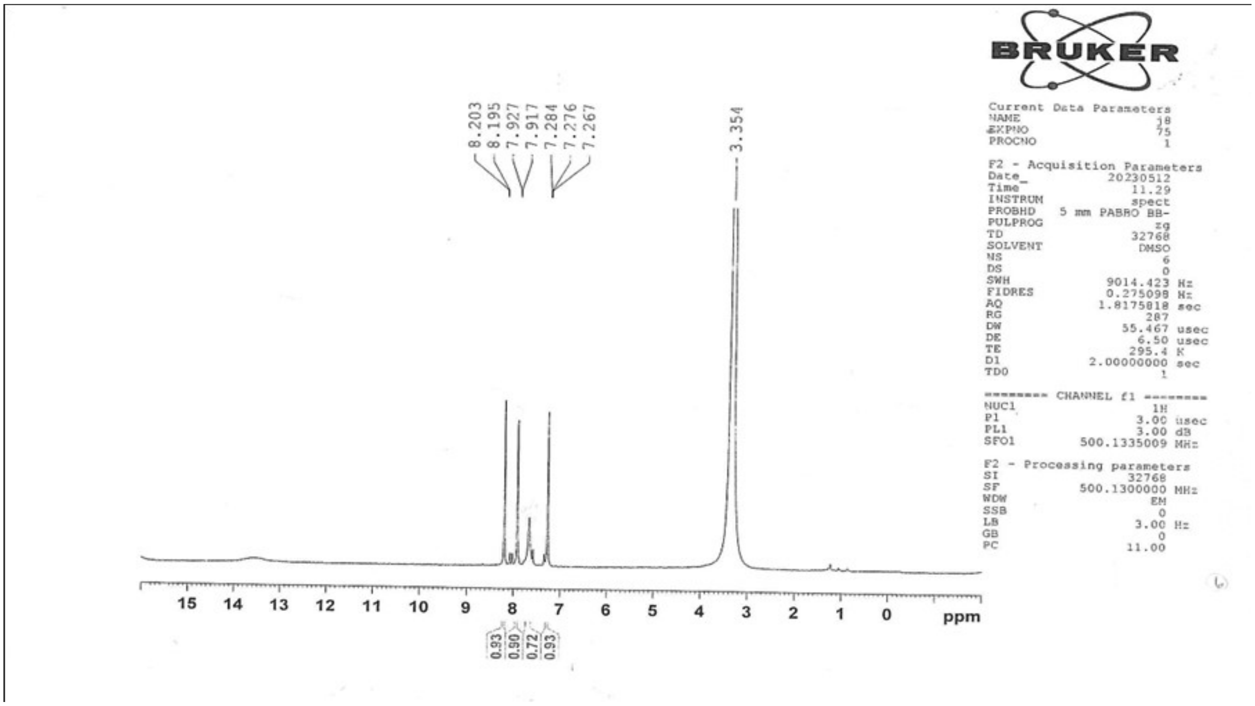

^{1}H NMR (500 MHz, DMSO-d_6) spectrum of (**1h**)

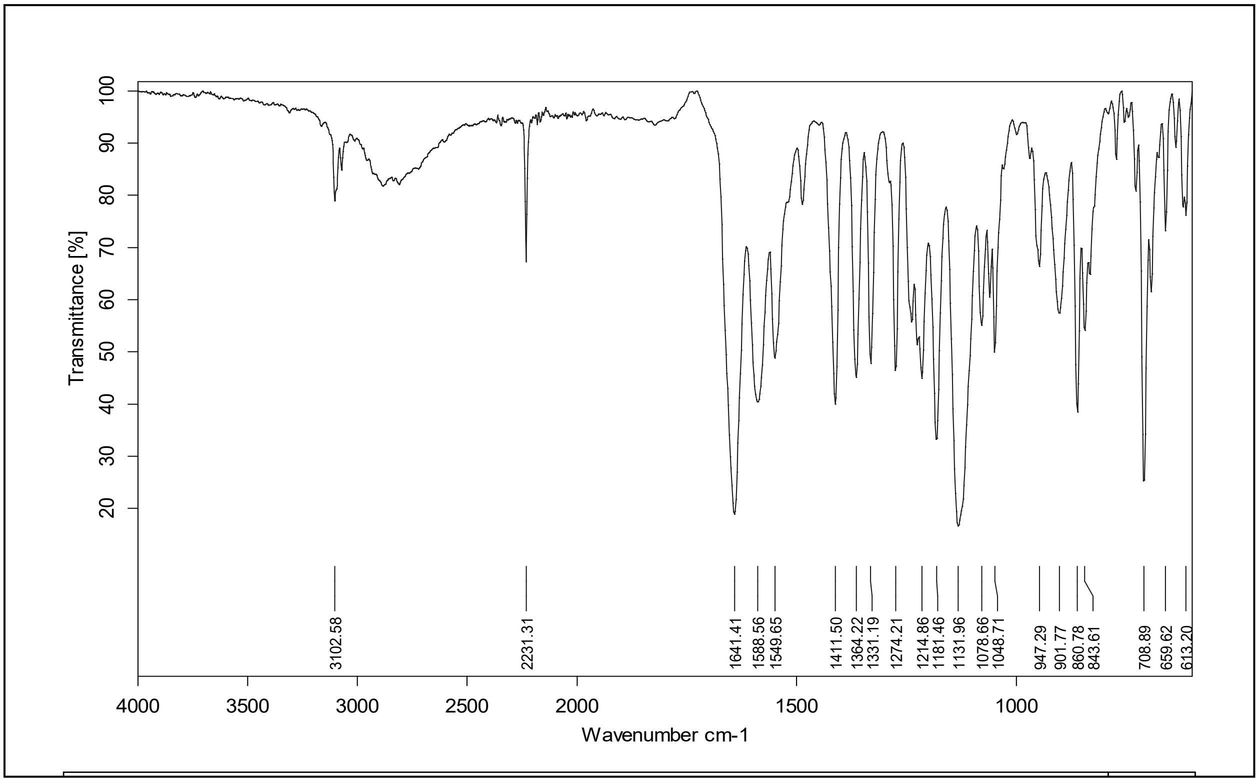

FT-IR (KBr) spectrum of (**1i**)

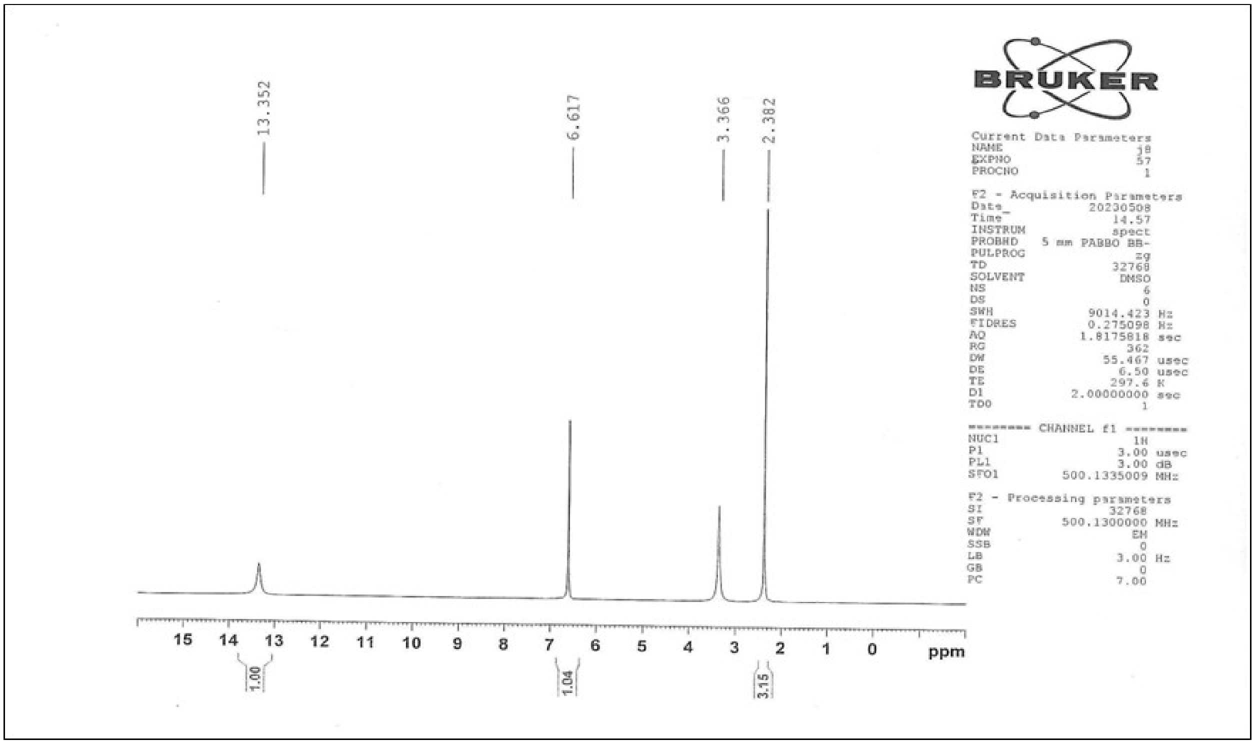

^{1}H NMR (500 MHz, DMSO-d_6) spectrum of (**1i**)

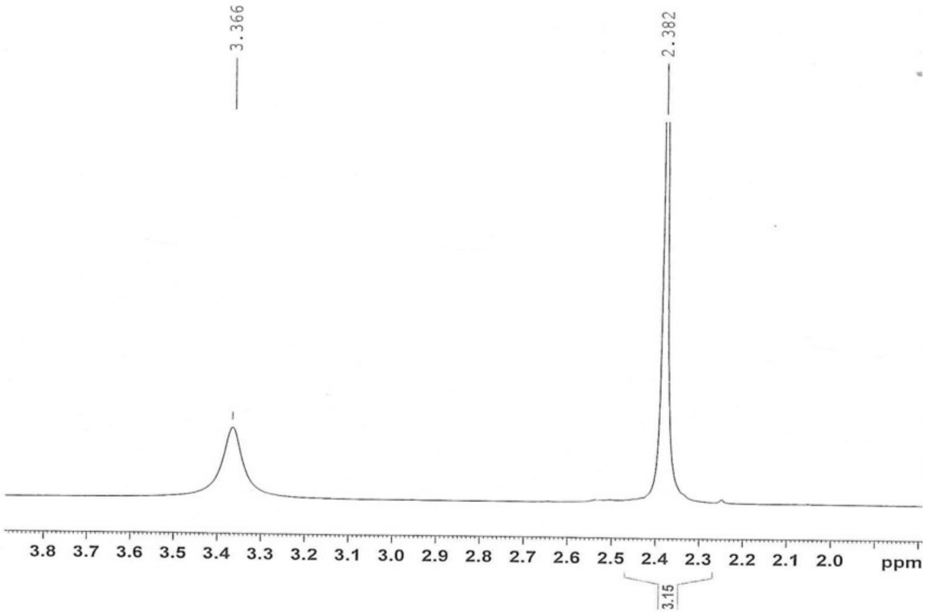

Expand of ^{1}H NMR (500 MHz, DMSO-d_6) spectrum of (**1i**)

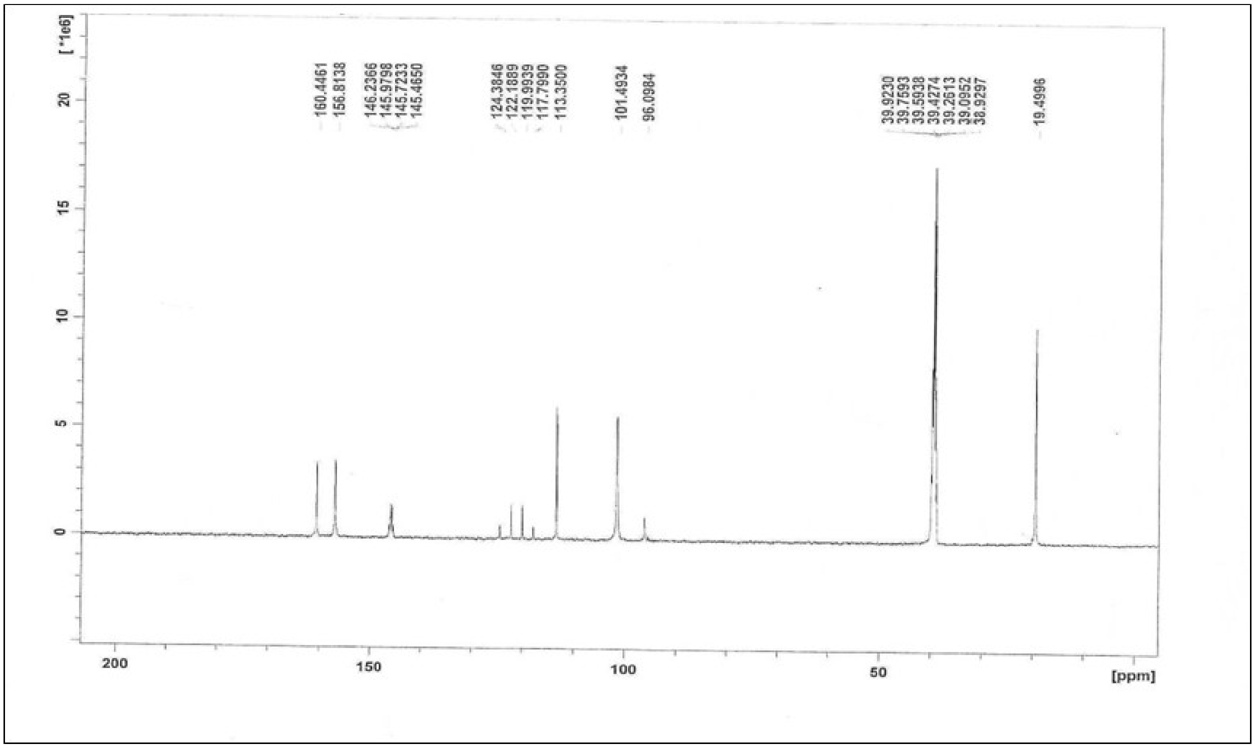

^{13}C NMR (125 MHz, DMSO-d_6) spectrum of (**1i**)

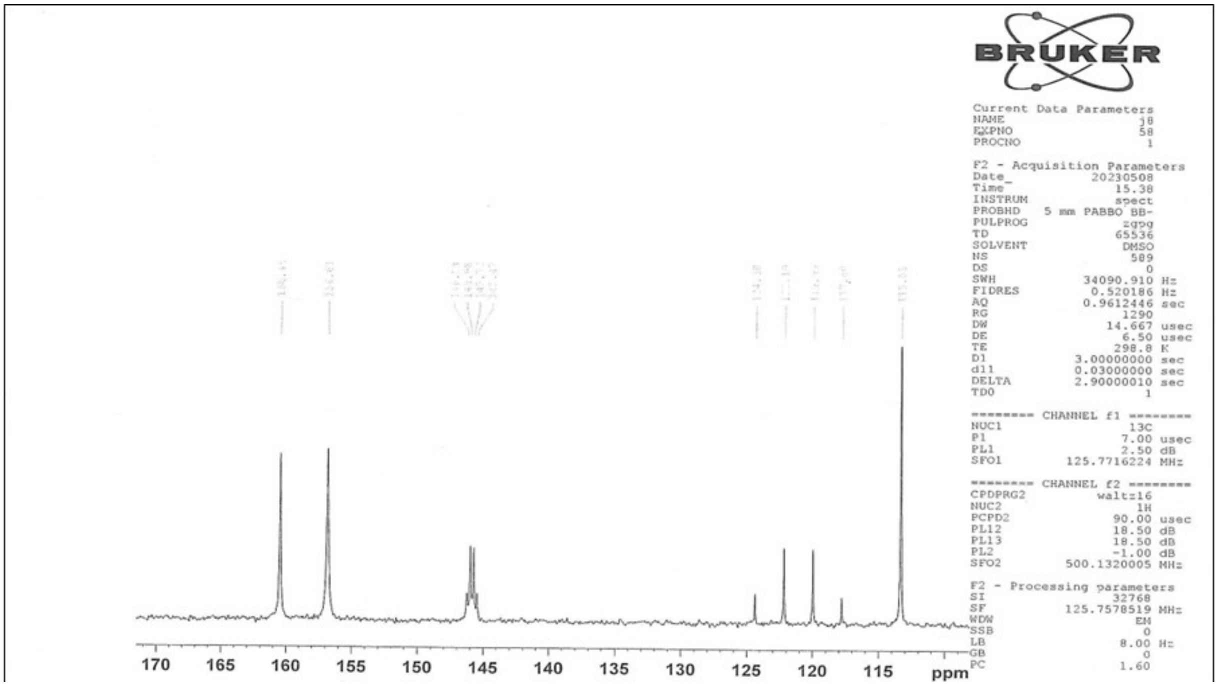

Expand of ^{13}C NMR (125 MHz, DMSO-d_6) spectrum of (**1i**)

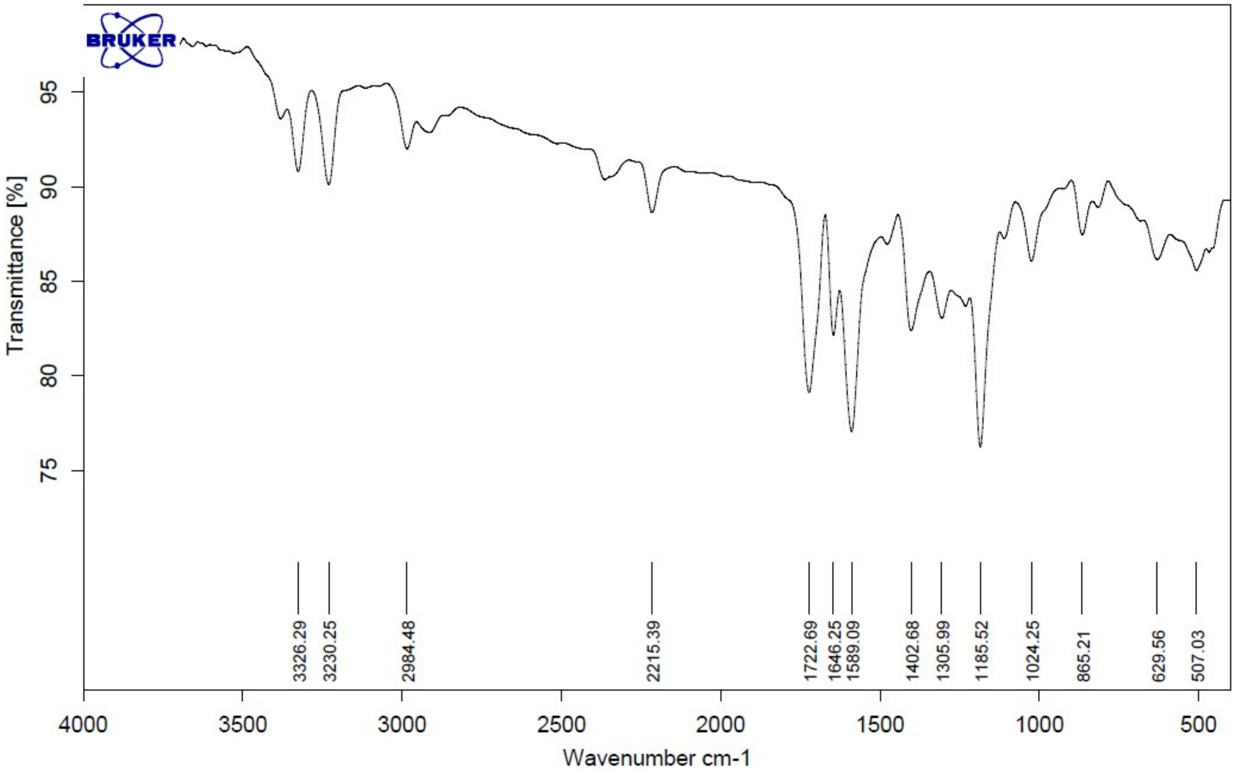

FT-IR (KBr) spectrum of (**2a**)

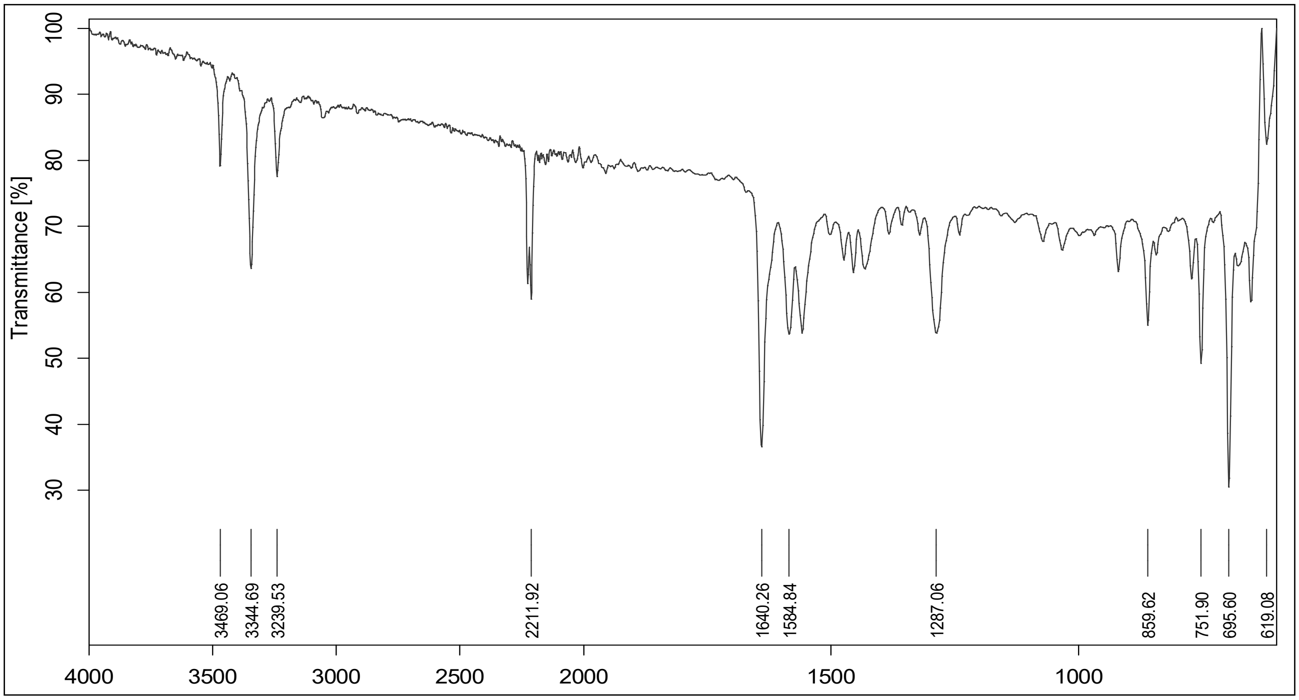

FT-IR (KBr) spectrum of (**2b**)

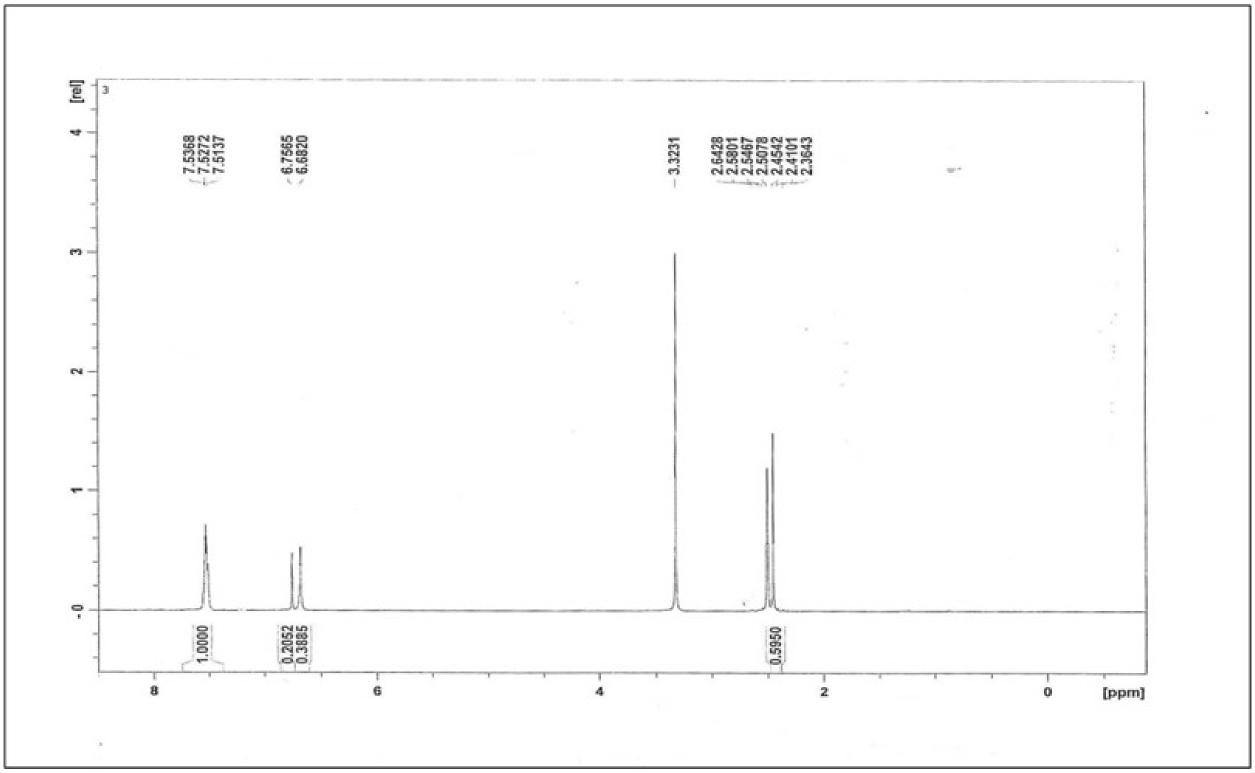

^{1}H NMR (500 MHz, DMSO-d_6) spectrum of (**2b**)

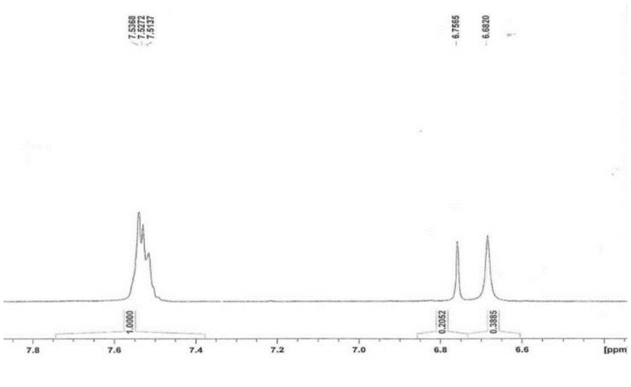

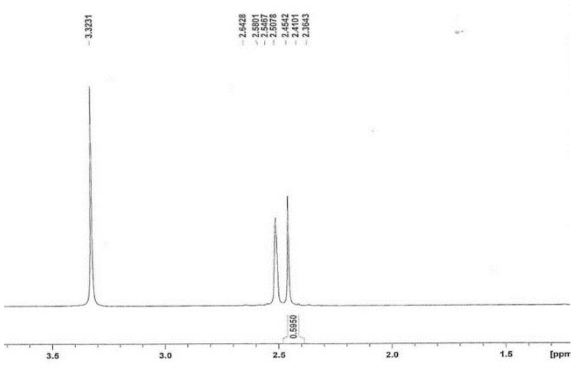

Expand of ^{1}H NMR (500 MHz, DMSO-d_6) spectrum of (**2b**)

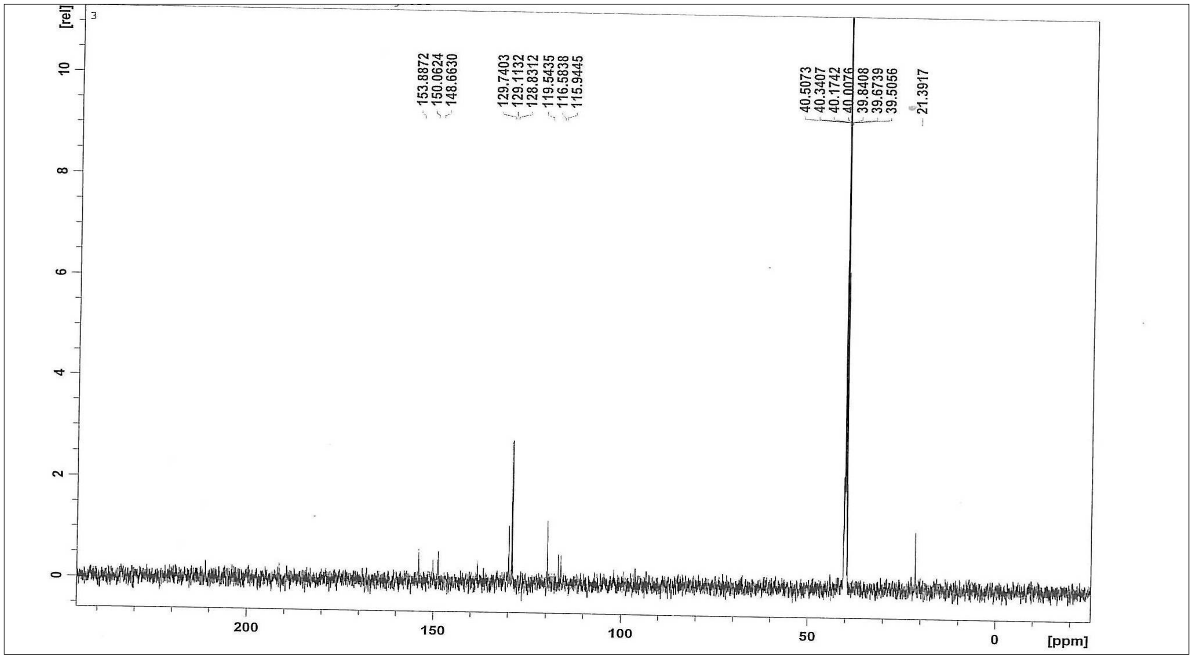

^{13}C NMR (125 MHz, DMSO-d_6) spectrum of (**2b**)

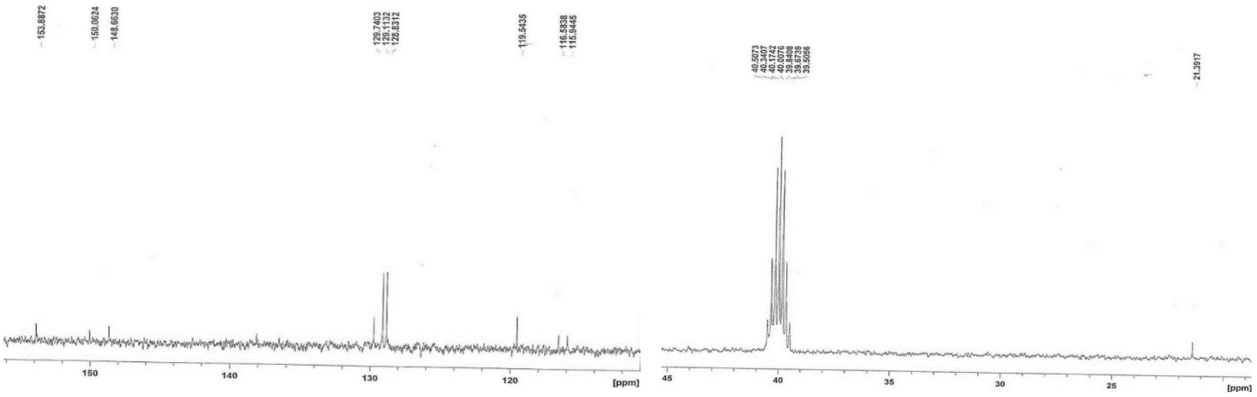

Expand of ^{13}C NMR (125 MHz, DMSO-d_6) spectrum of (**2b**)
